# Supplementary material for: Information-Theoretic Limits and One-Bit Estimation of Sparse Relation Matrices Under Personalized Local Differential Privacy and Byzantine Contamination
Source: Entropy (Basel). 2026 Oct 5;28(10):1088. doi: 10.3390/e28101088 (PMC13703432; doi:10.3390/e28101088)
Supplement: Supplementary file 1 [file entropy-28-01088-s001.zip › entropy-4532751-supplementary/Definitions/entropy-logo-eps-converted-to.pdf]

entropy
