# Supplementary material for: Information-Theoretic Limits and One-Bit Estimation of Sparse Relation Matrices Under Personalized Local Differential Privacy and Byzantine Contamination
Source: Entropy (Basel). 2026 Oct 5;28(10):1088. doi: 10.3390/e28101088 (PMC13703432; doi:10.3390/e28101088)
Supplement: Supplementary file 1 [file entropy-28-01088-s001.zip › entropy-4532751-supplementary/figures/fig_attacks.pdf]

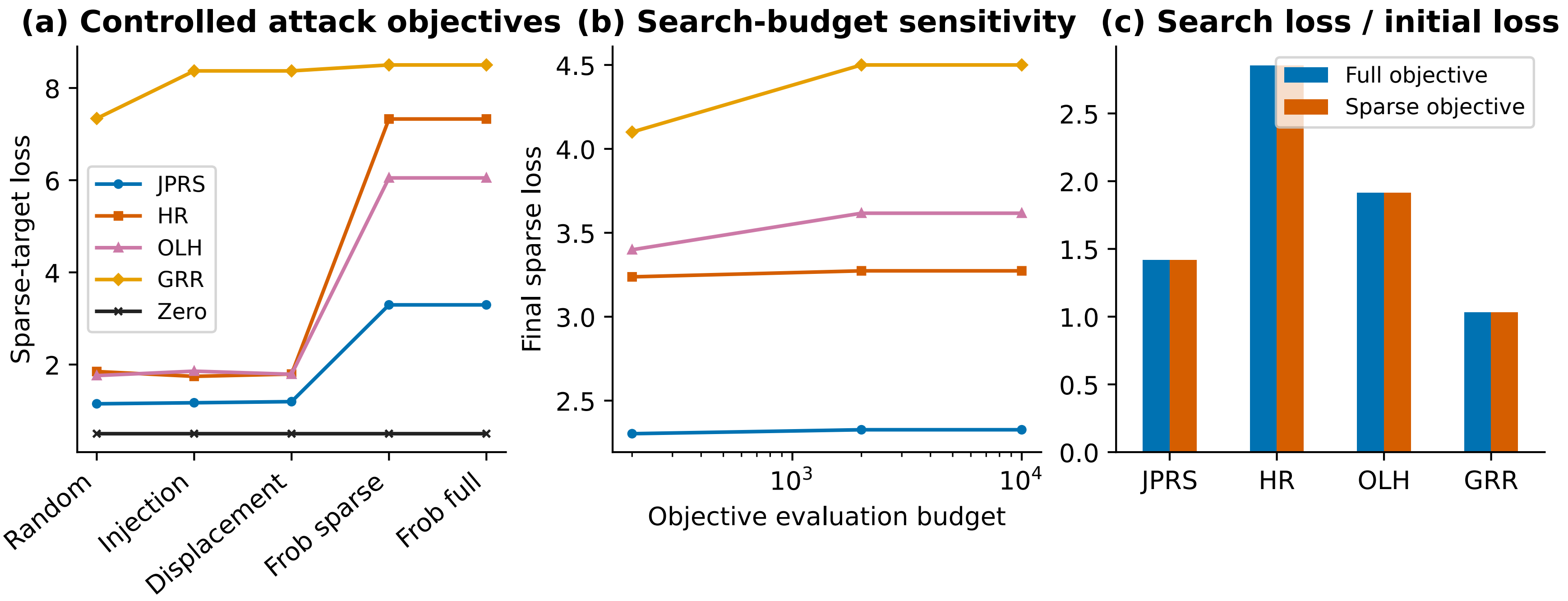

(a) Controlled attack objectives
(b) Search-budget sensitivity
(c) Search loss / initial loss
Sparse-target loss
JPRS
HR
OLH
GRR
Zero
Random
Injection
Displacement
Frob sparse
Frob full
Final sparse loss
Objective evaluation budget
10^3
10^4
Full objective
Sparse objective
JPRS
HR
OLH
GRR
