# Supplementary material for: Information-Theoretic Limits and One-Bit Estimation of Sparse Relation Matrices Under Personalized Local Differential Privacy and Byzantine Contamination
Source: Entropy (Basel). 2026 Oct 5;28(10):1088. doi: 10.3390/e28101088 (PMC13703432; doi:10.3390/e28101088)
Supplement: Supplementary file 1 [file entropy-28-01088-s001.zip › entropy-4532751-supplementary/figures/fig_diagnostics.pdf]

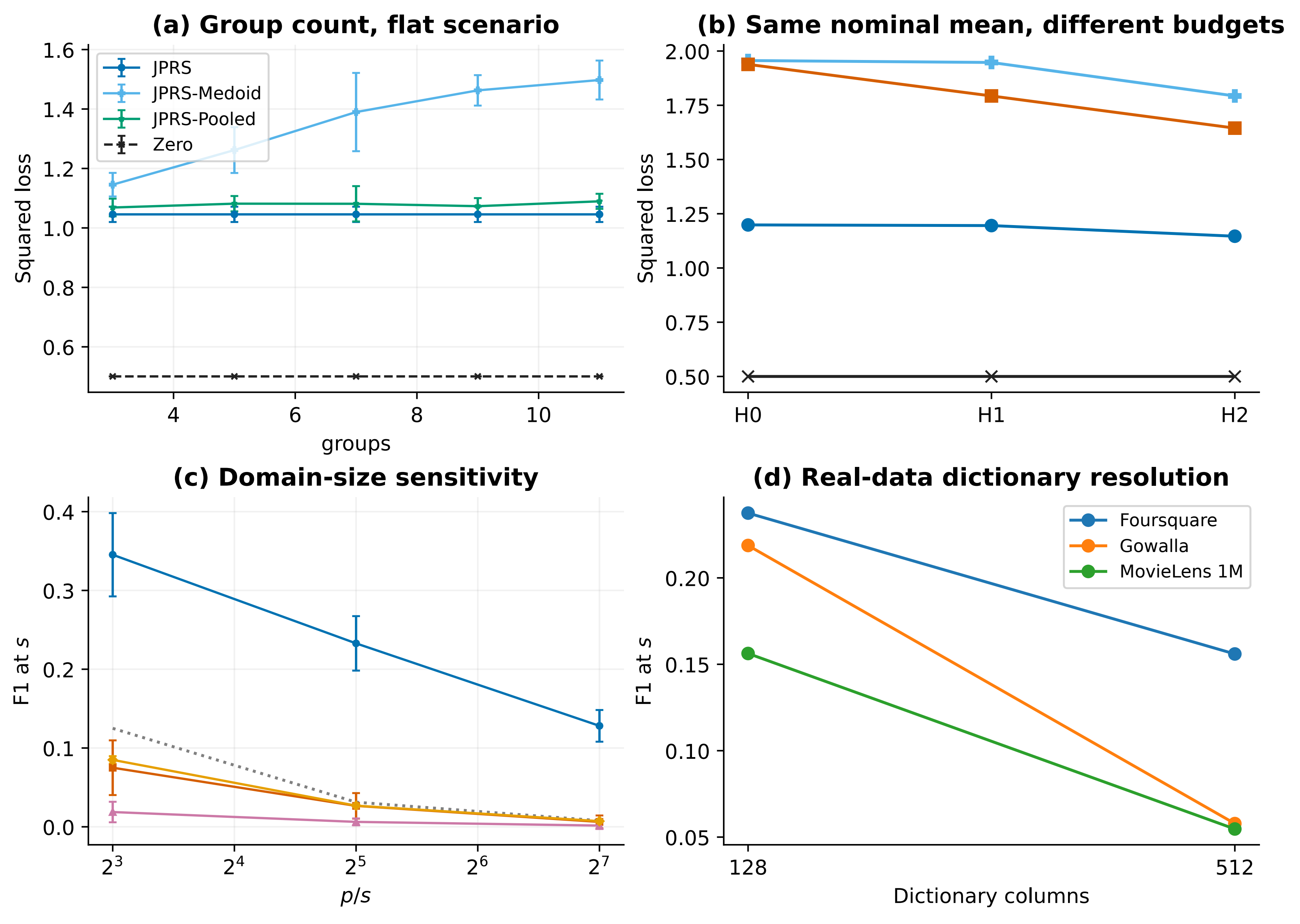
(a) Group count, flat scenario
JPRS
JPRS-Medoid
JPRS-Pooled
Zero
Squared loss
groups
(b) Same nominal mean, different budgets
Squared loss
H0
H1
H2
(c) Domain-size sensitivity
F1 at s
p/s
(d) Real-data dictionary resolution
Foursquare
Gowalla
MovieLens 1M
F1 at s
Dictionary columns
