# Supplementary material for: Information-Theoretic Limits and One-Bit Estimation of Sparse Relation Matrices Under Personalized Local Differential Privacy and Byzantine Contamination
Source: Entropy (Basel). 2026 Oct 5;28(10):1088. doi: 10.3390/e28101088 (PMC13703432; doi:10.3390/e28101088)
Supplement: Supplementary file 1 [file entropy-28-01088-s001.zip › entropy-4532751-supplementary/figures/fig_theory.pdf]

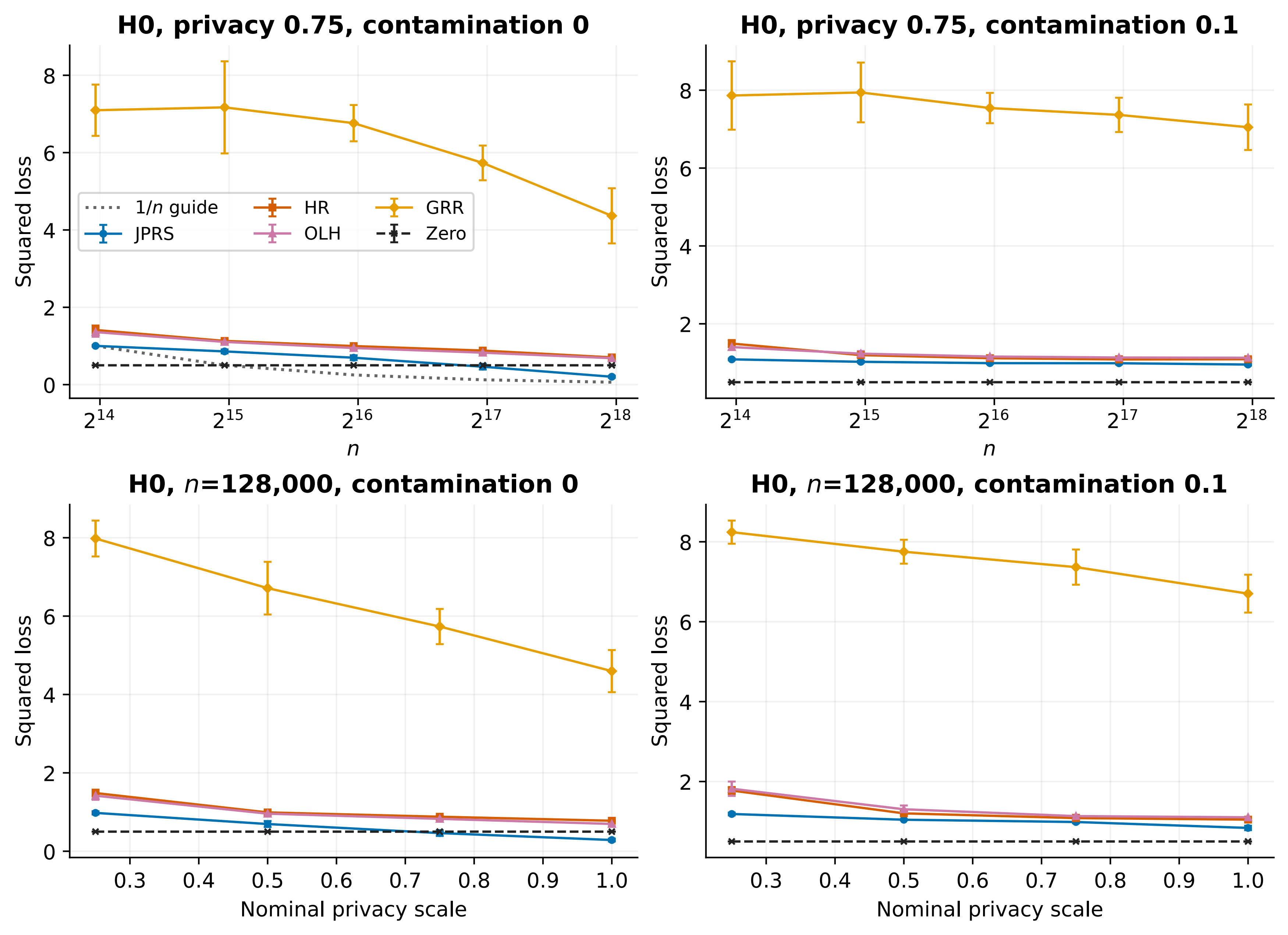
H0, privacy 0.75, contamination 0
H0, privacy 0.75, contamination 0.1
H0, n=128,000, contamination 0
H0, n=128,000, contamination 0.1
Squared loss
n
Nominal privacy scale
$1/n$ guide
JPRS
HR
OLH
GRR
Zero
2^{14}
2^{15}
2^{16}
2^{17}
2^{18}
0
2
4
6
8
0.3
0.4
0.5
0.6
0.7
0.8
0.9
1.0
