# Supplementary material for: Information-Theoretic Limits and One-Bit Estimation of Sparse Relation Matrices Under Personalized Local Differential Privacy and Byzantine Contamination
Source: Entropy (Basel). 2026 Oct 5;28(10):1088. doi: 10.3390/e28101088 (PMC13703432; doi:10.3390/e28101088)
Supplement: Supplementary file 1 [file entropy-28-01088-s001.zip › entropy-4532751-supplementary/Package_S1.zip!/Package_S1/publication/proof_checks/entropy_sparse_relation_manuscript.pdf]

entropy

Article

Information-Theoretic Limits and One-Bit Estimation of Sparse Relation Matrices Under Personalized Local Differential Privacy and Byzantine Contamination

Jia Ouyang [1] and Jinze Liu [2,*]

1 School of Low-Altitude Technology and Engineering, Guangdong Polytechnic Normal University, Guangzhou 510665, China; ouyangjia1@163.com
2 School of Arts & Sciences, University of Rochester, Rochester, NY 14627, USA
* Correspondence: liujinze9@gmail.com

Abstract

We study estimation of globally sparse relation probability matrices from capped binary relation sets under personalized user-level local differential privacy and Byzantine response replacement. A cardinality-calibrated joint personalized relation sketch (JPRS) releases one bit per user. The decoder combines public channel-amplitude weighting with exact sparse feasible projection. We distinguish trimmed honest information, adversarial leverage, and a response-contamination indistinguishability radius. An explicit capped binary block construction yields a sampling lower bound with an explicit low-information truncation. A separate contamination construction gives a finite-sample error floor. For precommitted corrupted identities, the global decoder admits a uniform bound against adaptive valid-output attacks. In the strong-privacy, sufficiently informative regime, sampling and contamination bounds match up to logarithms on bounded-ratio budget classes, subject to the stated sample regularity and corruption-count conditions. The theory uses global sparsity rather than low-rank structure. Across six synthetic families, the global decoder has lower mean loss than the three tested private global baselines, but improves on zero only in the Zipf setting. Three public-data benchmarks further show that low aggregate error can be driven by a merged tail category. These results delimit practical utility; finite-budget attack searches do not certify worst-case performance.

Keywords: personalized local differential privacy; sparse relation matrix; Byzantine contamination; minimax estimation; information-theoretic limits; one-bit sketch

Check for updates

Academic Editor: Firstname Lastname

Received:
Revised:
Accepted:
Published:

Copyright: © 2026 by the authors. Licensee MDPI, Basel, Switzerland. This article is an open access article distributed under the terms and conditions of the Creative Commons Attribution (CC BY) license.

1. Introduction

Many information systems record a small set of relations for each holder. Examples include rating-level–item pairs held by a user, activity–concept pairs held by a learner, and weekday–location pairs held by a visitor. The holder is the privacy unit; the two relation axes are shared attributes, not necessarily a holder identifier. If $\Omega = [d_1] \times [d_2]$, holder i contributes $E_i \subseteq \Omega$, while the server seeks the population relation probability matrix $M = \mathbb{E}A_i$, where A_i is the binary incidence matrix of E_i. Its entries are marginal probabilities and need not sum to one. Direct collection exposes a person's behavioral support. Local differential privacy (LDP) avoids a trusted curator by randomizing before transmission [1,2], but three features make this setting different from a standard frequency

oracle: one holder owns a set, privacy budgets can differ by identity, and compromised clients can emit strategically chosen valid reports.

An entrywise construction is not satisfactory. Perturbing all $p = d_1 d_2$ entries spends communication proportional to p; reporting only active indices exposes the private support; and splitting one user-level budget over the active relations loses utility with $|E_i|$. Related approaches address set-valued records [3], high-dimensional publishing [4], and multiple-sample sparse estimation [5]. Personalized mechanisms accommodate different user budgets [6,7], but these studies do not characterize what remains identifiable when an adversary occupies the most informative identities.

Response contamination is especially subtle under LDP. A forged output can look statistically ordinary because honest reports are already randomized; consequently, generic outlier removal has no reliable geometric signal. Existing work establishes that privacy and contamination can interact nonadditively [8,9], and attacks on frequency and key–value protocols demonstrate that syntactically valid outputs suffice to create large bias [10–12]. The relevant question is thus not only how much honest information survives, but how much signed influence corrupted channels retain after inversion.

We address this question for a globally sparse relation probability matrix. The joint personalized relation sketch (JPRS) computes one signed score over the entire capped relation set and releases one personalized randomized bit. The server forms a global budget-weighted matrix moment and performs an exact sparse capped-simplex projection. The proof uses the fact that a Byzantine identity can choose only one scalar sign multiplying its public pair-sign vector; it cannot choose an independent attack for each reconstructed coordinate. This output-domain coupling yields a global robust bound and makes grouped medoid selection unnecessary for the core theorem.

The scope is deliberately precise. The rectangular indices retain the semantics and output layout of a relation table, whereas the statistical class uses only global support sparsity. The rates therefore depend on $p = d_1 d_2$, not $d_1 + d_2$; no low-rank, row-sparse, graph-spectral, or smooth two-way structure is claimed. Joint processing protects the complete record in a single local channel; this design alone does not imply universal accuracy dominance over entrywise mechanisms. Global sparsity restricts how many marginal relations can be nonzero, whereas low rank restricts linear dependence and can hold for dense tables. Graph smoothness instead couples neighboring coordinates through a supplied graph. Neither low rank nor graph smoothness follows from our support bound. Real data with a nonzero tail are evaluated against both the full mean and its sparse projection, rather than assumed to belong exactly to the sparse model.

Figure 1 summarizes the reporting and reconstruction steps.

Our contributions are:

- a formal separation of edge, bounded-set, and user-level personalized LDP under authenticated response-level Byzantine contamination;
- a cardinality-calibrated one-bit relation-set mechanism with an exact linear matrix moment and weighting that minimizes a worst-case variance bound within the unbiased linear class;
- trimmed-information and linear-leverage functionals, communication-unrestricted lower bounds under the stated assumptions and a global JPRS upper bound, with matching up to logarithms restricted to the sufficiently informative regime and the explicit conditions in Proposition 2;
- a recomputed evaluation package with 30 frozen synthetic populations, three public datasets, bounded native-alphabet attack searches, shared feasible projection across global baselines, projection ablation, paired confidence intervals, and an explicit theorem-regime audit.

https://doi.org/10.3390/e1010000

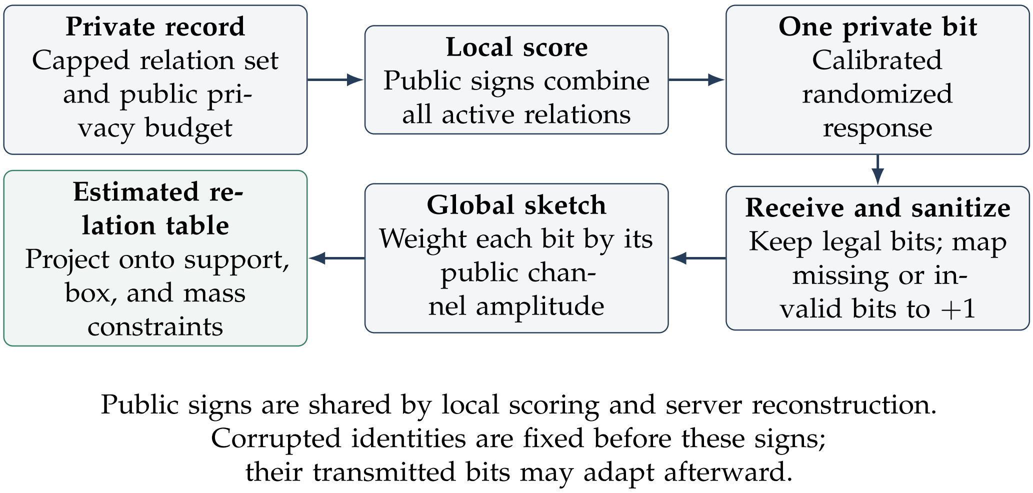

Figure 1. JPRS from the holder to the server. The upper row is local processing; the lower row is reconstruction. One corrupted identity controls one transmitted bit, which affects all coordinates through that identity's fixed public signs. The formal channel and decoder appear in Sections 5 and 6.

2. Related Work

Locally private estimation began with randomized response [1] and now has sharp minimax characterizations [13–15]; broader foundations and applications are reviewed in [2,16]. Communication-efficient frequency oracles include Randomized Aggregatable Privacy-Preserving Ordinal Response (RAPPOR), optimized local hashing (OLH), and Hadamard response (HR) [17–19]; Comparative evaluations [20] and locally private learned sketches [21] complement the general sketching foundations of Charikar et al. [22]. Sparse recovery supplies related projection ideas [23], although its observation model is not local privacy. Wang et al. [24] establish the $dk/(n\varepsilon^2)$ minimax scale for locally private numerical vectors, while Zhou et al. [25] give communication-efficient user- and event-level bounds for sparse vector aggregation. Neither studies personalized budgets or Byzantine response replacement. The closest multiple-sample result, Ma et al. [5], likewise does not combine those two complications. Sampling one relation and applying a native frequency oracle remains an important baseline here because it isolates the value of retaining the whole set.

Personalized local differential privacy (PLDP) assigns user-specific budgets, for example in frequency estimation and graph statistics [6,7]. Input-discriminative privacy [26] instead varies protection by input value. Set-valued LDP [3] addresses multiple items per holder. These formulations do not characterize sparse matrix risk under a worst-case budget-dependent corrupted set. Robust estimation [27], heavy-tailed mean estimation [28], Byzantine-robust learning [29], and studies combining privacy and robustness [8,9,30–32] provide related tools under different observation, trust, and output models. Poisoning attacks on LDP frequency and key–value protocols [10–12] motivate valid-output attacks, but do not supply the present pair-domain sparse-mean upper and lower bounds.

Table 1 compares assumptions rather than treating individual ingredients as new. Set-joint means that a channel protects a whole multi-relation record; personal budgets allow different privacy levels by identity; response contamination permits forged local outputs; global sparsity restricts the target support; and minimax denotes risk bounds over a specified model class. These properties are not interchangeable across observation units, losses, or adversary models. Duchi et al. [13] also study sparse means of potentially dense signed observations. Here, nonnegative binary records imply that $M_e = 0$ forces $A_i(e) = 0$ almost surely. Thus the observation models differ. "Sparse matrix" below means a globally sparse Cartesian table; low-rank geometry is outside our model.

https://doi.org/10.3390/e1010000

Table 1. Positioning against the closest methodological lines. Set-joint means that one user-level channel processes the complete capped set. **Y** indicates a studied feature, a triangle indicates a related feature under a different record or target model, and a dash means it is not addressed in that formulation. Minimax entries refer to each cited work's own loss and assumptions, not a common rate.

Work	Set-Joint	Personal Budgets	Response Byz.	Global Sparsity	Minimax	Scope Not Covered
Duchi et al. [13]	–	–	–	△	**Y**	Personalization and contamination
Zhou et al. [25]	**Y**	–	–	△	**Y**	Personalization and contamination
Ma et al. [5]	△	–	–	**Y**	**Y**	Personalization and contamination
Wu et al. [3]	**Y**	–	–	△	–	Sparse-risk theory and attacks
Chhor–Sentenac [9]	–	–	**Y**	–	**Y**	Personalized budgets and set-level privacy
Roy Chowdhury et al. [32]	△	–	**Y**	–	△	Personalized sparse-table rate
PLDP studies [6,7]	△	**Y**	–	–	–	Byzantine response contamination
This work	**Y**	**Y**	**Y**	**Y**	**Y**	Low rank and post-codebook compromise

The batch-contamination model of Chhor and Sentenac privatizes individual samples within a batch; it does not impose our privacy guarantee on replacing an entire capped set. Ma et al. study multiple-sample user-level sparse estimation, a related observation model rather than this binary relation channel.

3. Materials and Methods

3.1. Relational Observations and Privacy Units

For n authenticated holders indexed by $i \in [n] = \{1, \ldots, n\}$, let $\Omega = [d_1] \times [d_2]$, $p = d_1 d_2$, and identify a relation set $E_i \subseteq \Omega$ with $A_i \in \{0,1\}^{d_1 \times d_2}$. Each holder is capped at $\|A_i\|_0 \leq k$. Write $\mathcal{X}_k = \{A \in \{0,1\}^{d_1 \times d_2} : \|A\|_0 \leq k\}$, where k, s are integers with $1 \leq k, s \leq p$. Honest records are independent but need not be identically distributed:

$$A_i \sim P_i, \qquad \mathbb{E}_{P_i} A_i = M.$$

The common mean belongs to

$$\mathcal{M}_{s,k} = \{M \in [0,1]^{d_1 \times d_2} : \|M\|_0 \leq s,\ \|M\|_1 \leq k\},$$

and $P_i \in \mathcal{P}_k(M)$ denotes any capped record law with this mean. This distribution-free formulation includes fixed-cardinality, variable-size, long-tailed, block, hub, and latent-mixture populations. Frobenius loss is equivalently squared ℓ_2 loss after a fixed bijection between Ω and $[p]$.

Remark 1 (Matrix scope)**.** *Only global support sparsity and the set cap enter the rates. The theorem depends on d_1, d_2 through $p = d_1 d_2$; it does not exploit low rank, row/column sparsity, or a $d_1 + d_2$ parameterization. The matrix notation preserves relation semantics and the reconstructed output layout, while the public sign operates on an encoded relation pair.*

3.2. Privacy Units and Public Randomness

Let U collect the fresh public random seeds, independent of records, committed budgets, and the corrupted identity set. Edge adjacency changes one relation; q-set adjacency changes at most q; user adjacency allows any two capped sets.

Definition 1 (Personalized user-level LDP)**.** *Conditional on every public seed, user i's channel Q_i satisfies*

$$Q_i(B \mid A) \leq e^{\varepsilon_i} Q_i(B \mid A') \quad \textit{for all } A, A' \in \mathcal{X}_k$$

and measurable output events B. Edge and set privacy follow immediately; edge privacy alone composes by a factor at most $2k$ *for user adjacency.*

The protocol is noninteractive and each identity sends one report after its budget and authenticated identity are committed. The guarantee is for one release per identity; repeated participation requires composition of the per-release privacy budgets.

3.3. Response Contamination and Budget Functionals

An unknown set $\mathcal{B}$ of at most $m_\alpha = \lfloor \alpha n \rfloor$ identities is corrupted. It is fixed before the fresh public pair-sign seeds; corrupted report values may then depend on the public codebook and all honest reports. This permits coordinated adaptive valid-output attacks but excludes post-codebook identity compromise and Sybil creation. Let $\mathcal{A}_\alpha$ denote all such attacks, including the choice of the fixed identity set and adaptive report maps. The minimax risk is

$$R^\star = \inf_{\Pi,\widehat{M}} \sup_{M\in\mathcal{M}_{s,k}} \sup_{\{P_i\in\mathcal{P}_k(M)\}} \sup_{\mathcal{A}_\alpha} \mathbb{E}\|\widehat{M} - M\|_F^2.$$

The infimum ranges over noninteractive local channels Π satisfying the committed privacy vector and server estimators $\widehat{M}$; communication is unrestricted for this lower-bound benchmark. The expectation includes the records and all public, private, and adversarial randomization.

3.4. Information, Leverage, and Identifiability

Set $\beta_n = m_\alpha/n$ and $\bar{k} = \min(k,s)$. Write $\gamma_i = \tanh(\varepsilon_i/2)$, and let $\gamma_{(1)} \le \cdots \le \gamma_{(n)}$. The worst-case honest squared information and corrupted linear leverage are

$$\mathsf{I}_{\text{eff}}^{(\alpha)} = \sum_{j=1}^{n-m_\alpha} \gamma_{(j)}^2, \tag{1}$$

$$\mathsf{L}_{\text{eff}}^{(\alpha)} = \max_{|\mathcal{B}|\le m_\alpha} \frac{\sum_{i\in\mathcal{B}}\gamma_i}{\sum_{i\notin\mathcal{B}}\gamma_i^2}. \tag{2}$$

Set $\mathsf{L}_{\text{eff}}^{(\alpha)} = 0$ if $m_\alpha = 0$, and $\mathsf{L}_{\text{eff}}^{(\alpha)} = +\infty$ if $m_\alpha > 0$ and a denominator vanishes. Upper bounds require $\mathsf{I}_{\text{eff}}^{(\alpha)} > 0$. They cannot be replaced by an arithmetic mean budget: an attacker may capture the largest budgets while estimation is supported by the remaining squared amplitudes. A third functional, $\varrho_{\text{ind}}^{(\alpha)}$, is a protocol-independent separation certified by the per-user contamination-overlap construction, using half the corruption allowance before imposing its hard cap. It is defined by the finite budget program in Supplementary Section S3, rather than asserted to be the largest indistinguishable separation of every particular protocol.

Proposition 1 (Homogeneous specialization)**.** *If all* $\varepsilon_i = \varepsilon > 0$ *and* $\gamma = \tanh(\varepsilon/2)$*, then*

$$\mathsf{I}_{\text{eff}}^{(\alpha)} = (n-m_\alpha)\gamma^2, \qquad \mathsf{L}_{\text{eff}}^{(\alpha)} = \frac{m_\alpha}{(n-m_\alpha)\gamma}.$$

For positive $\varepsilon \to 0$*, with the exact fraction* $\beta_n = m_\alpha/n$*,*

$$\mathsf{I}_{\text{eff}}^{(\alpha)} = \frac{n(1-\beta_n)\varepsilon^2}{4}\{1+O(\varepsilon^2)\}, \qquad \mathsf{L}_{\text{eff}}^{(\alpha)} = \frac{2\beta_n}{(1-\beta_n)\varepsilon}\{1+O(\varepsilon^2)\}.$$

The leverage expansion is zero when $m_\alpha = 0$*.*

Complete adjacency relations, channel formalization, realizability, and the program defining $\varrho_{\mathrm{ind}}^{(\alpha)}$ are given in Supplementary Sections S1–S3.

4. Information-Theoretic Limits and Identifiability

Lemma 1 (Private contraction and contamination overlap). *An ε_i-LDP channel contracts total variation (TV) by at most $\gamma_i = \tanh(\varepsilon_i/2)$. Moreover, two output laws R_0, R_1 have intersecting β-contamination neighborhoods iff*

$$\mathrm{TV}(R_0, R_1) \le \frac{\beta}{1-\beta}.$$

Here $0 \le \beta < 1$, and a β-contamination neighborhood is $\{(1-\beta)R + \beta H : H \text{ is an output probability law}\}$. The first statement converts matrix separation into personalized testing information; the second converts a sufficiently small output separation into exact nonidentifiability.

Theorem 1 (Private sparse sampling lower bound). *Suppose $p \ge s$, $0 \le \alpha < 1/2$, and $\max_i \varepsilon_i \le 1$. For $b = \bar{k} = \min(k,s) \ge 1$, define*

$$B_{s,k}(I) = \frac{b}{1+I} \vee \min\left\{\frac{b^2}{s}, \frac{bs}{I}\right\}, \qquad bs/0 := +\infty. \tag{3}$$

There is an absolute constant $c > 0$ such that

$$R^\star \ge c B_{s,k}(\mathsf{I}_{\mathrm{eff}}^{(\alpha)}). \tag{4}$$

In particular the $bs/\mathsf{I}_{\mathrm{eff}}^{(\alpha)}$ lower bound holds when $\mathsf{I}_{\mathrm{eff}}^{(\alpha)} \ge s^2/b$. No matching low-information saturation claim is made. The two terms arise from different submodels: a correlated two-point problem and independent binary blocks. Their maximum is a lower bound, not an asserted exact risk curve.

Proof roadmap. Each record activates one binary coordinate in each of several independent blocks. Orthogonal likelihood scores and Bessel's inequality bound the sum of neighboring private divergences by each user's squared channel amplitude. Assouad's argument gives the second term in Equation (3); a correlated Bernoulli submodel gives the first. Corrupting the identities with largest amplitudes leaves information exactly $\mathsf{I}_{\mathrm{eff}}^{(\alpha)}$. Supplementary Section S4 provides the full construction and proof. □

Theorem 2 (Contamination indistinguishability). *With $m_\alpha = \lfloor \alpha n \rfloor$, there exists a personalized radius $\varrho_{\mathrm{ind}}^{(\alpha)} \in [0, 1/2]$ such that*

$$R^\star \ge \frac{\bar{k}(\varrho_{\mathrm{ind}}^{(\alpha)})^2}{16}\left(1 - 4e^{-m_\alpha/6}\right)_+. \tag{5}$$

The finite-count factor is strictly positive exactly when $m_\alpha \ge 9$; for $0 \le m_\alpha \le 8$, this inequality gives only zero. For homogeneous positive budgets,

$$\varrho_{\mathrm{ind}}^{(\alpha)} = \min\left\{\frac{1}{2}, \frac{m_\alpha}{(2n - m_\alpha)\gamma}\right\}.$$

If $2m_\alpha \ge n$, then $R^\star \ge \bar{k}/4$ for every privacy vector.

Proof roadmap. Construct two means differing on $\bar{k}$ coordinates. Private contraction ensures per-user overlap. Independent corruption indicators chosen before the public seed give identical transcript laws before enforcing the count cap; conditioning on at most m_α corrupted identities yields the displayed finite-count factor. The majority case uses identical transcript laws obtained by swapping candidate honest sets. See Supplementary Section S3. □

Corollary 1 (Necessary identifiability conditions)**.** *In the strong-privacy regime, consistency requires*

$$B_{s,k}(\mathsf{l}_{\mathrm{eff}}^{(\alpha)}) \to 0, \qquad \bar{k}(\varrho_{\mathrm{ind}}^{(\alpha)})^2(1-4e^{-m_\alpha/6})_+ \to 0, \qquad 2m_\alpha < n \textit{ eventually}. \tag{6}$$

For homogeneous budgets and fixed k, s, these imply $\mathsf{l}_{\mathrm{eff}}^{(\alpha)} \to \infty$ and $\beta_n/\gamma \to 0$ if $m_\alpha \geq 9$ eventually. When $\varepsilon \to 0$, the latter is $\beta_n = o(\varepsilon)$. For growing s, k, use Equation (6) rather than an unconditional $\mathsf{l}_{\mathrm{eff}}^{(\alpha)} \gg \bar{k}s$ criterion.

The contamination lower bound allows arbitrary finite budgets; the sampling bound is restricted to $\max_i \varepsilon_i \leq 1$. Throughout, c, C, C_0 denote positive absolute constants that may differ between statements, unless dependence on α_0, C_γ is explicitly stated. The contraction and neighborhood-intersection facts are classical [9,14,27]; the proofs in Supplementary Section S2 are included to specify the conventions.

5. Joint Personalized Relation Sketch

Encode each relation pair by a public bijection $\iota : \Omega \to [p]$. For identity i, a public finite-wise independent hash generates $R_i(e) \in \{-1, 1\}$; signs are independent across identities and jointly independent on every set of at most $\min\{p, k+2s+1\}$ queried pairs. A finite-wise independent hash can evaluate signs on demand without storing a length-p vector; the stated independence is a mathematical requirement and must be checked for any concrete hash implementation.

For $m \geq 1$, let

$$a_m = m^{-1}\mathbb{E}\left|\sum_{j=1}^{m} \xi_j\right|, \quad \kappa_k = \sqrt{k}a_k, \quad c_k(m) = a_k/a_m, \quad c_k(0) = 0,$$

where ξ_j are independent Rademacher variables ($\Pr(\xi_j = \pm 1) = 1/2$). The sequence a_m is nonincreasing, so $0 < c_k(m) \leq 1$ for $1 \leq m \leq k$, and $2^{-1/2} \leq \kappa_k \leq 1$. Given A_i, compute

$$\theta_i(A_i) = c_k(\|A_i\|_0)\ \mathrm{sgn}_0\left(\sum_{e \in E_i} R_i(e)\right)$$

where $\mathrm{sgn}_0(0) = 0$ and $\mathrm{sgn}_0(t) = \mathrm{sgn}(t)$ otherwise. Marginalizing an independent private fair tie coin gives exactly this same output law. An empty record releases a fair bit and remains in the public denominator. Then send one bit

$$\Pr(Y_i = y \mid A_i, U) = \frac{1 + y\gamma_i\theta_i(A_i)}{2}. \tag{7}$$

Algorithm 1. Local JPRS randomizer

1. Evaluate $R_i(e)$ only for the at most k pairs in E_i.
2. Form the cardinality-calibrated set score $\theta_i(A_i)$.
3. Draw $Y_i \in \{-1, 1\}$ from Equation (7) and release only Y_i.

https://doi.org/10.3390/e1010000

The private payload is one bit and local work is $O(k)$. The score is a single joint function of the complete set, not an entrywise composition.

Theorem 3 (Privacy and calibrated moment)**.** *The channel satisfies user-level ε_i-LDP conditional on every public seed, and*

$$\mathbb{E}[\theta_i(A_i)R_i(e)] = \frac{\kappa_k}{\sqrt{k}}M_e. \tag{8}$$

Consequently, for any honest set J, $D_J = \sum_{i\in J}\gamma_i^2$,

$$\widetilde{M}_J(e) = \frac{\sqrt{k}}{\kappa_k D_J}\sum_{i\in J}\gamma_i Y_i R_i(e)$$

is unbiased and has coordinate variance at most $k/(\kappa_k^2 D_J) \le 2k/D_J$, for $D_J > 0$.

Proof roadmap. The output likelihood ratio is bounded by $(1+\gamma_i)/(1-\gamma_i) = e^{\varepsilon_i}$. Exchangeability of the active Rademacher signs and the definition of $c_k(m)$ give the exact first moment; weighted Cauchy–Schwarz minimizes the worst-case coordinate variance bound within the unbiased linear class. The constant 2 bounds $1/\kappa_k^2$; it is not generally equal to it. See Supplementary Section S5. No row–column factor is required for the theorem; the pair-safe sign is used precisely to avoid unsupported claims of low-rank matrix geometry. □

6. Budget-Aware Byzantine-Robust Decoding

Legal bits are retained; every missing or malformed packet is mapped to $+1$ by a fixed deterministic sanitizer. Sending malformed data therefore gives the attacker no action beyond the already legal bit $+1$. Put $D = \sum_{i=1}^n \gamma_i^2$ and form the observable global sketch

$$V(e) = \frac{\sqrt{k}}{\kappa_k D}\sum_{i=1}^{n}\gamma_i \overline{Y}_i R_i(e). \tag{9}$$

Figure 1 locates this sketch in the full procedure. Define the mathematical decoder using an exact Euclidean minimizer,

$$\mathcal{P}_{s,k}(V) \in \arg\min_{X\in\mathcal{M}_{s,k}} \|V - X\|_F^2.$$

If $D = 0$, return zero. Otherwise select at most s positive coordinates of the original V in descending value, breaking ties by increasing coordinate index. On this set S, return

$$\widehat{M}_j = \min\{1, \max\{0, V_j - \lambda\}\} \quad (j \in S), \qquad \widehat{M}_j = 0 \quad (j \notin S).$$

Use $\lambda = 0$ if the clipped selected mass is at most k; otherwise choose $\lambda \ge 0$ so that the mass equals k. The threshold acts on the original values, not pre-clipped values. Supplementary Section S5 proves this characterization. The revised routine solves it to floating-point tolerance, with independent optimality tests; the statistical theorem is stated for the mathematical exact minimizer.

Algorithm 2. Global JPRS decoder

1. Sanitize every received packet to $\overline{Y}_i \in \{-1, 1\}$.
2. Accumulate the budget-weighted pair sketch in Equation (9).
3. Return $\widehat{M} = \mathcal{P}_{s,k}(V)$.

The global denominator is public and includes every committed identity; no attacker-dependent honest-set denominator is used. For fixed s, k, the decoder uses public budgets and signs and does not require α. The analysis uses α. The optional, unimplemented unknown-sparsity rule in Supplementary Section S6 instead requires a known upper bound on contamination.

Put $q_\star = \min(2s, p)$. For $1 \le q \le p$, define

$$T_q(\delta) = q \log(9ep/q) + \log(8/\delta), \qquad \gamma_{\max} = \max_i \gamma_i.$$

The upper-bound regularity condition is

$$k \le s, \qquad \mathsf{I}_{\mathrm{eff}}^{(\alpha)} > 0, \qquad \mathsf{I}_{\mathrm{eff}}^{(\alpha)} \ge C_0 \gamma_{\max}^2 T_{q_\star}(\delta), \tag{10}$$

and the corrupted identity set is fixed before the fresh pair-sign codebook. Unlike the sampling lower bound, the decoder theorem does not require $\max_i \varepsilon_i \le 1$.

Theorem 4 (Global robust JPRS recovery)**.** *Suppose Equation (10) holds and $\alpha < 1/2$. Uniformly over the model and adaptive valid-output attacks, Algorithm 2 satisfies with probability at least $1 - \delta$,*

$$\|\widehat{M} - M\|_F^2 \le Ck\left[\frac{s\log(9ep/s) + \log(8/\delta)}{\mathsf{I}_{\mathrm{eff}}^{(\alpha)}} + (\mathsf{L}_{\mathrm{eff}}^{(\alpha)})^2\right]. \tag{11}$$

Proof roadmap. Replace forged reports by independent honest ghost bits. The resulting global sketch is unbiased and concentrates uniformly on every $2s$-coordinate comparison set. Conditional on the public codebook, a sparse random-matrix operator bound is uniform over all adaptive Byzantine sign choices; its linear term is $\sum_{i \in \mathcal{B}} \gamma_i$, while the mixed term is absorbed by Equation (10). Exact feasible projection converts the sparse directional event to Frobenius loss. Supplementary Section S5 gives the full net argument and all event allocations. Intuitively, more honest squared amplitude reduces sampling uncertainty, whereas corrupted linear amplitude survives channel inversion as bias; projection restricts the comparison to at most $2s$ coordinates. □

Corollary 2 (Expected risk)**.** *Let $\mathcal{R}(\Pi_{\mathrm{JPRS}})$ denote the worst-case expected squared Frobenius loss of the fixed JPRS protocol, with the same suprema as in the minimax definition. For $n \ge 2$ and Equation (10) evaluated at $\delta = n^{-2}$,*

$$\mathcal{R}(\Pi_{\mathrm{JPRS}}) \le C\left[\bar{k} \wedge k\left\{\frac{s\log(9epn)}{\mathsf{I}_{\mathrm{eff}}^{(\alpha)}} + (\mathsf{L}_{\mathrm{eff}}^{(\alpha)})^2\right\}\right] + \frac{2\bar{k}}{n^2}. \tag{12}$$

Proposition 2 (A sufficient regular-budget class)**.** *Fix $\alpha \le \alpha_0 < 1/2$ and suppose $0 < g = \gamma_{\min} \le \gamma_i \le C_\gamma g$. With $\beta_n = m_\alpha/n$, constants depending only on α_0, C_γ give*

$$\mathsf{L}_{\mathrm{eff}}^{(\alpha)} \asymp \beta_n/g, \qquad \min\{1, \mathsf{L}_{\mathrm{eff}}^{(\alpha)}\} \asymp \varrho_{\mathrm{ind}}^{(\alpha)} \asymp \min\{1, \beta_n/g\}.$$

All quantities are zero when $m_\alpha = 0$. In addition, assume $k \le s$, $\max_i \varepsilon_i \le 1$, $\mathsf{I}_{\mathrm{eff}}^{(\alpha)} \ge s^2/k$, Equation (10) at $\delta = n^{-2}$, and either $m_\alpha = 0$ or $m_\alpha \ge 9$. Then the sampling and contamination lower bounds match the expected upper bound up to $\log(epn)$. This does not assert matching outside these conditions.

https://doi.org/10.3390/e1010000

For homogeneous positive budgets, a sufficient condition for vanishing upper risk is

$$\frac{ks\log(epn)}{n(1-\beta_n)\gamma^2} \to 0, \qquad k\left\{\frac{\beta_n}{(1-\beta_n)\gamma}\right\}^2 \to 0,$$

subject to the regularity condition. Replacing γ by $\varepsilon/2 + O(\varepsilon^3)$ gives factors 4 in the sampling term and 4 in the squared contamination term before the universal theorem constant. These are sufficient conditions; general necessity is stated separately in Corollary 1.

Computation and Scope

The server forms the global vector in $O(np)$ signed operations. The implemented stable sort and fixed-precision threshold search add $O(p\log p)$ work, using $O(p)$ memory; local work and private payload are $O(k)$ and one bit. We do not claim sublinear exact decoding. Grouped medoid and central-pooling reconstructions are retained only as optional empirical operating points and deterministic stability results in the supplement; they do not improve the proved minimax rate under the fixed-before-codebook threat model. Matching has all the restrictions in Proposition 2, including the information threshold and corruption-count condition. Arbitrary heterogeneous-budget matching, post-codebook compromise, Sybil attacks, low-rank structure, and sublinear decoding remain open.

7. Experimental Results

7.1. Design, Data, and Evaluation

All numerical results use the implementation with corrected sparse projection, zero-information decoding, separate public/private random streams, and the pairwise-uniform OLH hash family. The fixed schedule contains 895 configurations and 12,875 method-level records. Five seeds are used for each parameter sweep; the primary six-scenario endpoint uses 20 paired seeds fixed before inspecting the revised results. Thirty supplied synthetic populations remain frozen, and the additional endpoint populations are reproducible from the released generator and seed list. The six families are flat, Zipf, community, hub–tail, user mixture, and variable cardinality. Controlled sweeps use the analytic uniform-support mean; the six-family benchmarks use the empirical mean of each frozen or generated holder population.

Unless varied, the synthetic setup has $n = 16{,}000$, $p = 64^2$, $s = 128$, $k = 8$, and seven groups for optional decoders. H0 uses equal budgets; H1 uses balanced multipliers $(0.75, 1.25)$ and H2 uses $(0.2, 1.8)$, shuffled across identities, with $\varepsilon_i = \min(2, \varepsilon_0 r_i)$. The CSV reports realized budget ranges and means. Corrupted identities have the largest public budgets and are fixed before public signs or hashes. The primary endpoint uses H1, $\varepsilon_0 = 1$, and $\alpha = 0.1$; this is a finite-sample benchmark, not a claim that all strong-privacy matching conditions hold. Unless explicitly varied, contaminated results use support displacement: each corrupted identity chooses a legal report maximizing its contribution to a linear false-minus-true top-s objective in the raw global estimate. Thus Tables 2 and 3 and Figures 2–5 use this attack whenever $\alpha > 0$; the $\alpha = 0$ curves are clean. Frobenius-loss searches are separate comparisons.

The experiments use the official MovieLens 1M [33] (https://grouplens.org/datasets/movielens/1m/ (accessed on 3 October 2026)), Foursquare NYC–Tokyo [34] (https://sites.google.com/site/yangdingqi/home/foursquare-dataset (accessed on 3 October 2026)), and Gowalla [35] (https://snap.stanford.edu/data/loc-gowalla.html (accessed on 3 October 2026)) archives; all three SHA-256 hashes agree with the recorded source manifest. Deterministic preprocessing reproduced all six processed files byte for byte. Eligible holder counts are 6040, 2293, and 107,092; each Gowalla run samples 32,000 holders without replacement, while its target remains the complete processed population mean. Relation

rows encode rating level, city, and UTC weekday, respectively. The main dictionary has 512 columns with $s = 128$; the resolution check uses 128 columns with $s = 32$. Duplicate relations are removed, the tail maps to an "other" column, and seeded minimum-hash caps each set at $k = 8$. Dictionaries are computed from the public source and treated as fixed benchmark side information; their private discovery is outside the privacy guarantee. Real targets need not be exactly s-sparse, so these are approximation benchmarks rather than direct tests of the sparse minimax theorem.

Padded relation sampling (P-RS) chooses uniformly from a record padded to k entries with a dummy symbol. We combine it with a public-coin Hadamard sign response (P-RS-HR), optimized local hashing (P-RS-OLH), or generalized randomized response (P-RS-GRR). The HR implementation is a public-row, one-bit variant; it is not the original private-coin HR protocol with its native communication and runtime guarantees. Supplementary Section S7 specifies these channels and their gain-weighted decoders. Grouped medoid/pooling branches are optional controls. The nonprivate top-s projection is an information reference. We additionally report the zero estimator and the data-independent uniform vector k/p; the latter is a dense reference and is not asserted to satisfy the sparse output constraint. Private mechanisms in a paired configuration use the same records, public budgets, seed index, and corruption placement. The nonprivate reference observes uncorrupted sampled records; the constant references need no records.

Let M_{pop} denote the full target and $M^{(s)}_{\text{pop}} = \mathcal{P}_{s,k}(M_{\text{pop}})$. We report sparse-target loss, complete-target loss, and normalized Frobenius error (NFE), respectively:

$$L_s = \|\widehat{M} - M^{(s)}_{\text{pop}}\|_F^2, \qquad L_{\text{full}} = \|\widehat{M} - M_{\text{pop}}\|_F^2, \qquad \text{NFE} = L_{\text{full}}/\|M_{\text{pop}}\|_F^2.$$

The zero reference has losses $\|M^{(s)}_{\text{pop}}\|_F^2$ and $\|M_{\text{pop}}\|_F^2$, making $L_s/L_{s,0}$ directly interpretable. Ranking-based F1 at s averages uniform boundary-tie resolution in both the estimated and true top-s lists. If their inclusion weights are u_j, v_j, this score is $s^{-1}\sum_j u_j v_j$. Its random-ranking chance level is s/p; an all-zero estimate therefore has chance ranking performance, not genuine support recovery. The deterministic tie metric is retained only for audit in the CSV. Chance is the expectation under uniform random ranking, not a lower bound on F1. Support displacement explicitly promotes false coordinates and suppresses true top-s coordinates, so attacked rankings can fall below chance. Tie averaging removes arbitrary boundary choices, not this adversarial ranking bias.

Error bars are pointwise 95% Student-t confidence intervals (CIs) across seeds, not standard deviations or simultaneous bands. Primary paired differences compare JPRS-Global with HR, OLH, GRR, zero, and uniform in each of six scenarios. We report paired mean-difference intervals and exact two-sided sign tests, with Holm adjustment across all 30 comparisons. The sign test concerns win probability; it is not a test of the magnitude of the mean difference. Five-seed secondary comparisons are descriptive; exact sign-flip probabilities have a minimum two-sided value of $2/2^5 = 0.0625$ when all differences have one sign. Intervals with only five seeds are approximate and should not be read as high-precision evidence.

7.2. Controlled Scaling and the Strong-Privacy Diagnostic

Figure 2 shows sample-size, privacy, and contamination sweeps together with the zero reference. These curves distinguish lower loss among private reconstructions from improvement over a data-independent estimate. We use the complete prespecified sample-size range to estimate log–log slopes, fit separately within each seed, and summarize the five slopes; we do not select a favorable tail after seeing the curve.

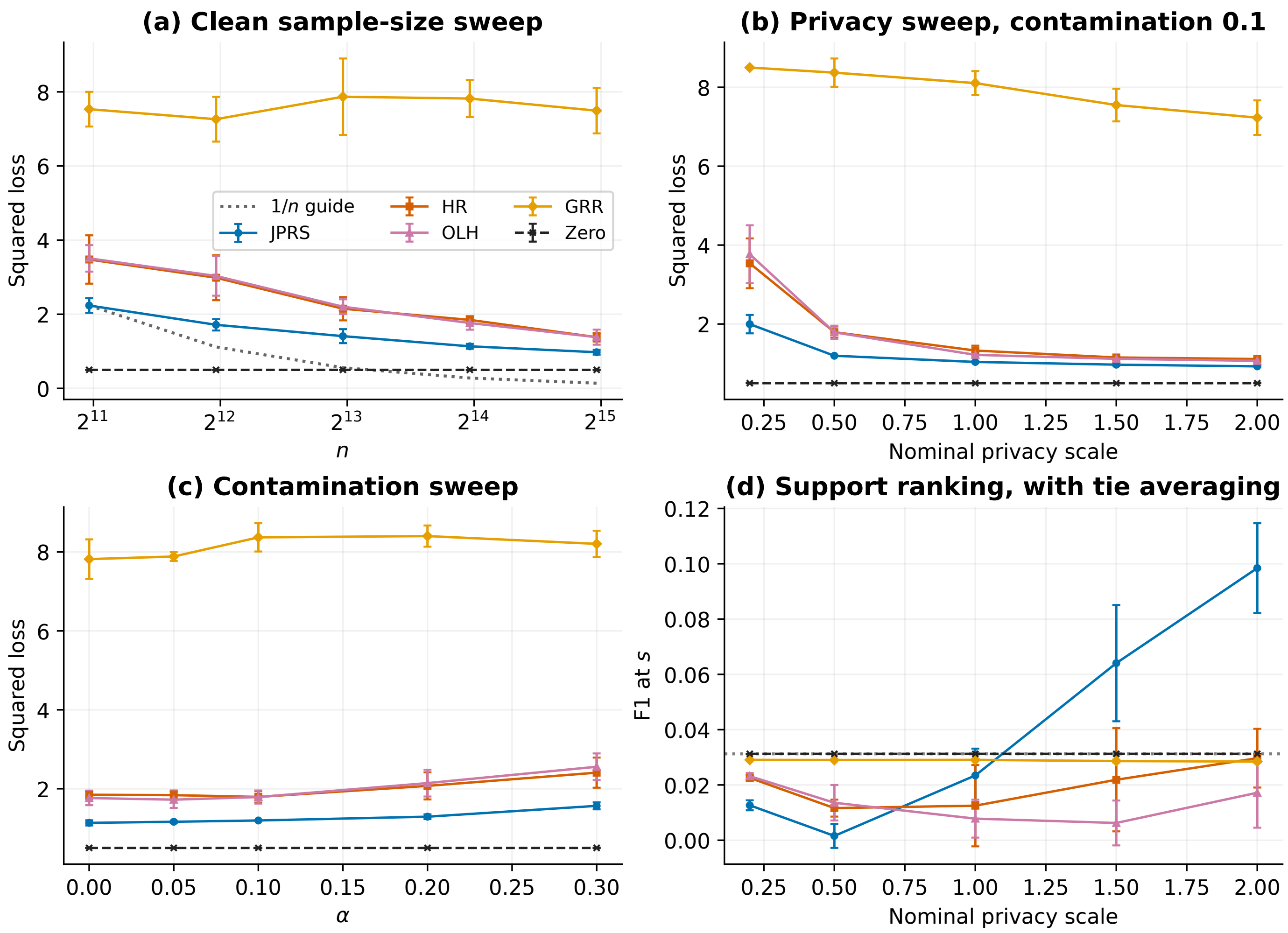

Figure 2. Controlled sweeps with five paired seeds. Error bars are pointwise 95% t intervals for the mean. Contaminated points use support displacement; $\alpha = 0$ is clean. H1 is used throughout, with nominal privacy 0.5 in (**a**,**c**), and contamination 0.1 in (**b**,**d**). The zero estimator and random-ranking chance level expose low-information behavior. The dotted $1/n$ guide in (**a**) is anchored at the first JPRS mean; it is a visual reference, not a fitted or asserted risk law.

The separate H0 audit sets $\varepsilon_0 \leq 1$, $p = 4096$, $s = 128$, and $k = 8$, with $n \in \{16{,}000, 32{,}000, 64{,}000, 128{,}000, 256{,}000\}$ and contamination 0 or 0.1 (Figure 3). It records both honest information and the threshold $\mathsf{I}_{\mathrm{eff}}^{(\alpha)} \geq s^2/k$. This threshold and strong privacy are necessary parts of the matching statement; they do not certify the unspecified universal constant in the theorem's separate regularity condition. The fitted clean JPRS slopes over all five points are -0.299 in the low-information H1 sweep, -1.384 in the nominal-privacy-2 H1 sweep, and -0.551 in the H0 audit. The nominal-privacy-2 curve is appreciably curved: successive doubling-interval slopes from $n = 16{,}000$ to 256,000, calculated within each seed and then averaged, are -0.543, -1.038, -1.634, and -2.369. Thus -1.384 summarizes this finite range, not a stable power-law exponent or an asymptotic rate (Supplementary Section S7.4). A risk upper bound need not be attained with equality, and these empirical slopes neither prove nor refute the bound by themselves. Paired contaminated minus clean losses, including negative increments if they occur, are retained for every matching configuration in the analysis files.

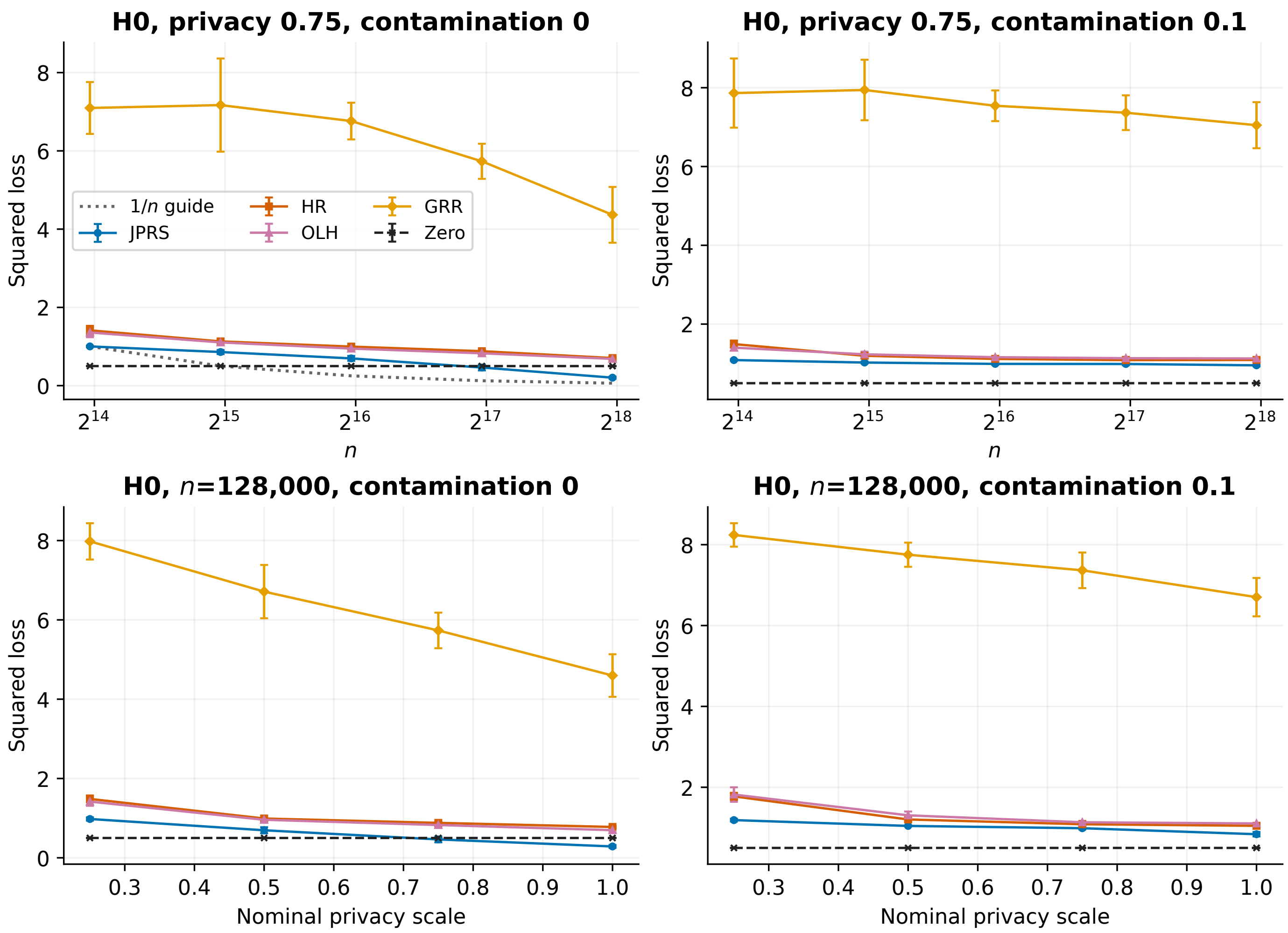

Figure 3. H0 strong-privacy diagnostics: means over five paired seeds with pointwise 95% Student-t confidence intervals for the mean. Clean and support-displacement-contaminated curves use matching configurations. All sample-size points enter the reported slope fits. The clean sample-size panel includes the same first-point-anchored $1/n$ guide as the controlled sweep.

7.3. Six Synthetic Families and Projection Ablations

Table 2 reports the revised 20-seed endpoint. JPRS-Global has lower mean loss than each of HR, OLH, and GRR in 6 of six families. It improves on the zero estimator in 1 of six; its mean-loss ratio to zero ranges from 0.369 to 2.058. Thus a favorable rank among the tested private methods does not support a blanket practical-utility claim. The paired estimates and all 30 adjusted tests appear in the supplementary paired-comparison table. All 30 Holm-adjusted sign tests are below 0.05, but their directions differ: 20 comparisons favor JPRS and 10 favor the constant reference. These tests do not justify a uniform superiority claim. At this endpoint, Equations (1) and (2) give $\mathsf{I}_{\text{eff}}^{(\alpha)} = 2995.878$ and $\mathsf{L}_{\text{eff}}^{(\alpha)} = 0.296194$. In Corollary 2, the sampling and contamination expressions before the unspecified constant are respectively 7.245 and 0.702, totaling 7.946, near the trivial risk scale $\bar{k} = 8$. The observed mean zero losses are only 0.383–2.656. The bound therefore supplies no quantitative guarantee of improvement over zero here; the five unfavorable comparisons with zero are consistent with that limitation, not a contradiction of a promised gain. This is a scale diagnostic, not an impossibility result: regularity is not certified for the unspecified C_0, and $\max_i \varepsilon_i = 1.25$ exceeds the strong-privacy range for matching, although $\mathsf{I}_{\text{eff}}^{(\alpha)} > s^2/k = 2048$. Supplementary Section S7.4 gives the calculation.

Table 2. Mean squared loss at nominal privacy 1 under support displacement with contamination 0.1 and 20 paired seeds. Zero and uniform are data-independent references.

Scenario	JPRS	HR	OLH	GRR	Zero	Uniform
Flat	1.03	1.291	1.273	7.926	0.5005	0.4849
Zipf	0.9801	2.133	2.032	10.09	2.656	2.641
Community	1.021	1.291	1.266	7.929	0.5005	0.4849
Hub-tail	1.151	1.451	1.424	8.105	0.6742	0.6586
Mixture	1.053	1.337	1.296	7.955	0.5512	0.5356
Variable	0.7519	1.157	1.095	7.717	0.3829	0.383

The corrected ablation compares the raw global sketch, box clipping alone, top-s plus box clipping, and full support/box/mass projection while holding the reports fixed. It therefore isolates the step used in the proof. The old medoid "NoProj" control was already box-clipped; it is now accurately named Medoid-ClipOnly and is excluded from claims about removing all postprocessing. The supplementary projection-ablation table gives absolute losses so that a large raw-to-projected ratio is not confused with improvement over zero. Figure 4(**d**) therefore uses zero loss as its denominator and retains the full projection alongside the partial projections.

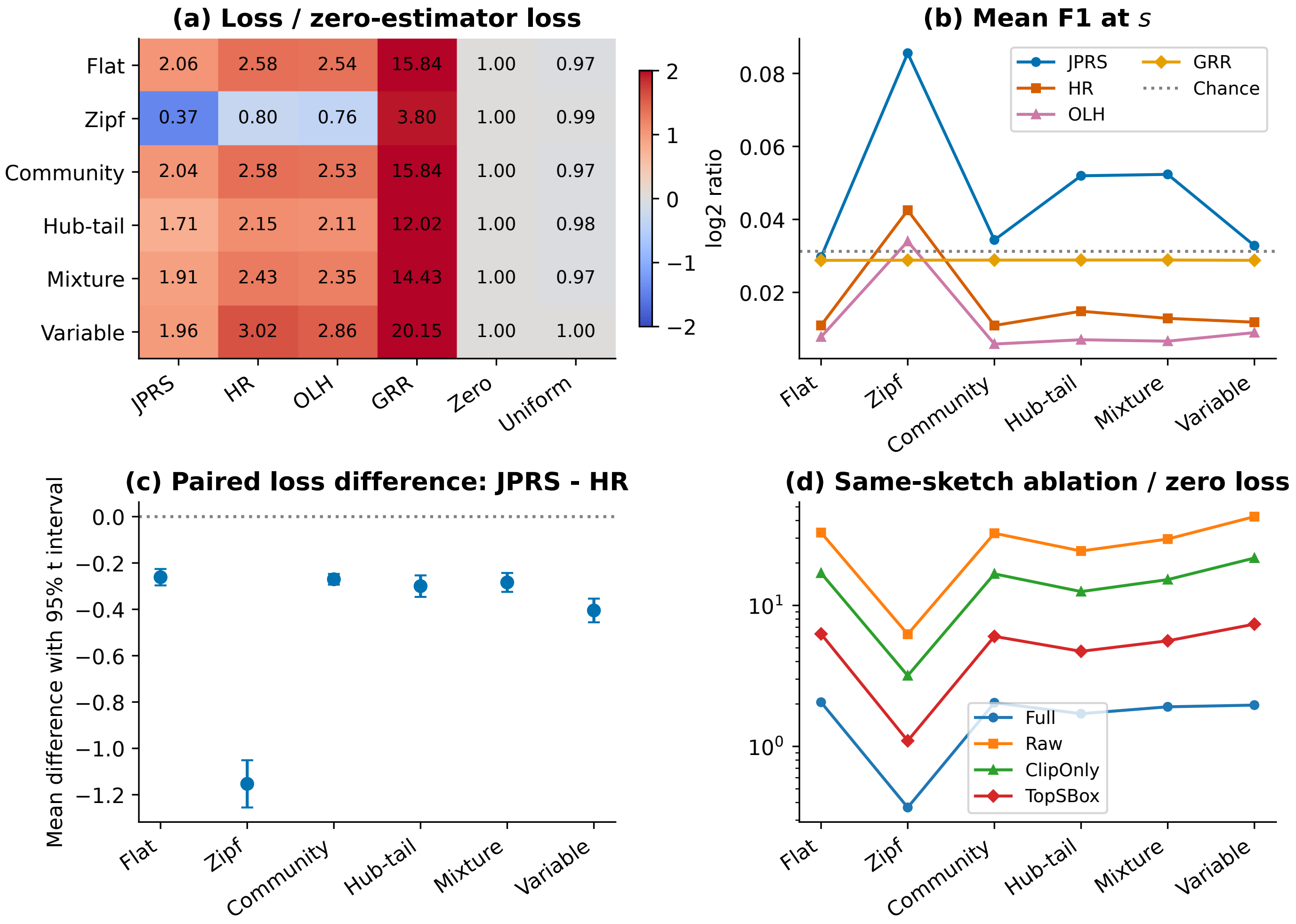

Figure 4. Twenty-seed synthetic endpoint under support displacement ($\alpha = 0.1$). (**a**) Mean loss divided by the zero reference, with log-scale color. (**b**) Tie-averaged support ranking. (**c**) Paired JPRS-minus-HR loss differences with pointwise 95% intervals. (**d**) Ablations of the same global raw sketch, divided by zero-estimator loss. Panels (**a**,**b**,**d**) show means without dispersion; panel (**c**) shows pointwise intervals.

7.4. Real-Data Benchmarks and Attack Objectives

Figure 5 and Table 3 report the recomputed real-data comparisons. At nominal privacy 2, JPRS-to-zero sparse-loss ratios are 2.069, 1.247, and 0.106 for MovieLens, Foursquare, and Gowalla, respectively. Full-target loss and NFE remain visible because sparse-target performance can hide approximation error. Chance F1 levels are 0.05, 0.125, and approximately 0.0357; the different row dimensions make a common absolute F1 value misleading across datasets. The compact-resolution results are in Supplementary Section S7.

An exploratory concentration audit found that 99.97% of Gowalla's complete target squared norm lies in the public "other" columns at this resolution. We therefore additionally report every constant $c \in \{0, 0.25, 0.5, 0.75, 1\}$ placed on those columns, with zeros elsewhere. This post hoc diagnostic is outside the primary 30 tests and does not select a tuned constant. The fixed $c = 0.5$ and $c = 0.75$ references attain sparse losses 0.232 and 0.094, respectively, below JPRS's 0.331. Thus improvement over zero on Gowalla does not establish recovery of specific-location relations or superiority to simple public-structure references. All constant losses appear in the supplementary exploratory table; both resolutions are retained in the analysis CSV.

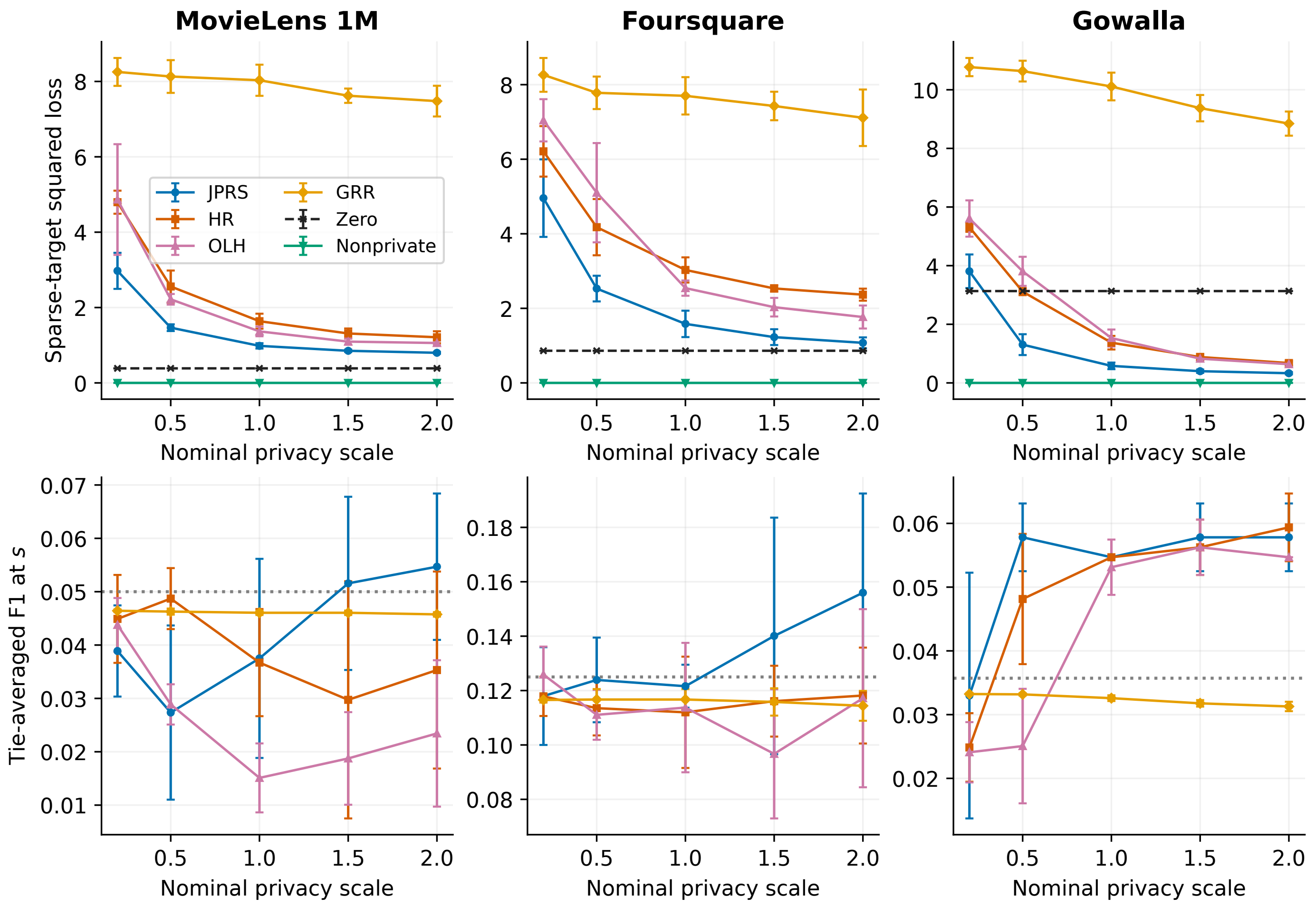

Figure 5. Real-data privacy sweeps under support displacement at contamination 0.1 and 512 dictionary columns. Points are means over five paired seeds; error bars are pointwise 95% Student-*t* confidence intervals for the mean. Dashed/dotted references show zero loss and the appropriate chance support-ranking score.

Support displacement maximizes a linear false-minus-true-support objective and is not a worst-case Frobenius attack. We therefore add native-alphabet searches for L_s and L_{full} after global feasible projection. The evaluation adversary knows the benchmark target and

sees the honest aggregate and public randomness, but its corrupted identity set is already fixed. This oracle knowledge belongs to the attack evaluation and is not supplied to the private decoder. Searches start from the displacement transcript, enumerate small spaces when possible, and otherwise use bounded multi-start coordinate search. We randomize candidate-label order to avoid privileging low-index labels when a budget ends partway through a large alphabet. The main search budget is 2000 objective evaluations, with 200 and 10,000 checked on a separate smaller domain.

Table 3. Real-data endpoint at nominal privacy 2 under support displacement with contamination 0.1 and dictionary size 512. Entries are means over five paired seeds. NFE uses the complete empirical population mean. F1 averages boundary ties.

Dataset	Method	Sparse Loss	Full Loss	NFE	F1
Foursquare	JPRS	1.075	1.068	1.223	0.156
Foursquare	Nonprivate	0	0.01022	0.01171	1
Foursquare	GRR	7.112	7.119	8.158	0.1144
Foursquare	HR	2.367	2.353	2.696	0.1182
Foursquare	OLH	1.767	1.757	2.013	0.1172
Foursquare	Zero	0.8624	0.8727	1	0.125
Gowalla	JPRS	0.3305	0.3292	0.1051	0.05781
Gowalla	Nonprivate	9.557×10^{-5}	0.0004371	0.0001395	0.9281
Gowalla	GRR	8.846	8.847	2.824	0.03128
Gowalla	HR	0.6781	0.6764	0.2159	0.05937
Gowalla	OLH	0.6387	0.638	0.2036	0.05469
Gowalla	Zero	3.133	3.133	1	0.03571
MovieLens 1M	JPRS	0.7991	0.7956	1.964	0.05469
MovieLens 1M	Nonprivate	0	0.01893	0.04674	0.9948
MovieLens 1M	GRR	7.478	7.497	18.51	0.04575
MovieLens 1M	HR	1.211	1.211	2.989	0.03532
MovieLens 1M	OLH	1.059	1.058	2.612	0.02344
MovieLens 1M	Zero	0.3862	0.4051	1	0.05

Every search records its initial/final loss, evaluation count, visited coordinates, completed sweeps, alphabet size, and budget exhaustion. Only an exhaustive search certifies optimality; equal evaluation budgets do not ensure equal optimization quality across alphabets. Medoid, pooled, and ablation outputs receive the same forged transcript but are not independently maximized. These experiments measure specified attacks, not the full adversarial supremum. Detailed search diagnostics and the sequential runtime audit are provided in Supplementary Section S7 and the reproducibility package. The 460 Frobenius-search calls contain no exhaustive optimum certificates; 410 exhaust their declared evaluation budget. The final scientific schedule has no failed rows, and interrupted attempts are retained separately.

8. Discussion

8.1. What the Theory Establishes

Two different budget summaries are unavoidable. Squared channel amplitudes govern honest testing information, whereas a valid forged bit enters linearly after channel inversion. Trimming the former and maximizing the latter over the same corruption budget explains why an arithmetic mean privacy budget is not sufficient. The indistinguishability radius adds a separate statement: even infinite computation cannot recover two targets whose personalized contamination neighborhoods intersect.

Under fixed-before-codebook identity corruption, the random pair signs couple all coordinates affected by one forged bit. A uniform operator-norm event controls every

adaptive legal sign simultaneously, so splitting users into groups is not needed for the rate. Using the global sketch avoids the group-size reduction in honest information. Medoid aggregation remains an optional decoder whose clean loss and attack increment must be compared in the relevant setting; the present theorem does not establish a statistical advantage for grouping.

8.2. What the Matrix Formulation Does Not Establish

The method jointly privatizes relation sets and reconstructs a rectangular table, but the proof sees an encoded Cartesian alphabet of size $p = d_1 d_2$. Consequently, the present paper should not be read as low-rank matrix completion, graphon estimation, or recovery with a $d_1 + d_2$ sample complexity. Incorporating row/column sharing, low rank, or graph smoothness without destroying user-level LDP and valid-output robustness is a separate problem.

8.3. Threats to Validity and Open Problems

The lower sampling bound assumes $\max_i \varepsilon_i \leq 1$; unrestricted large-budget matching is open. The strongest matching statement also requires sufficient information, the corruption-count condition, sample regularity, and a bounded-ratio budget class, while arbitrary heterogeneous vectors may leave a gap between $\mathsf{L}_{\mathrm{eff}}^{(\alpha)}$ and $\varrho_{\mathrm{ind}}^{(\alpha)}$. Identities are authenticated and fixed before the fresh public codebook; post-codebook compromise and Sybil creation would invalidate the signed-output argument. Exact decoding scans all p relations. Real-data targets depend on public preprocessing, and the five-seed sweeps and 20-seed endpoints across six synthetic scenarios cannot establish universal empirical dominance. Gowalla is dominated by the public other columns, and some fixed public-structure references outperform JPRS; a low overall loss does not establish specific-location recovery. Finally, the experiments simulate the reporting layer; device energy, peak memory, network loss, and end-to-end deployment latency are not measured.

These limitations suggest four concrete directions: low-rank or row/column-structured pair sketches, sublinear candidate generation with a privacy-safe public dictionary, protocols robust to post-codebook compromise, and adaptive decoders that estimate unknown sparsity and contamination without requiring a known upper bound on α.

9. Conclusions

The one-bit JPRS mechanism protects entire capped relation sets under personalized user-level local privacy. The analysis separates honest squared information, corrupted linear leverage, and contamination nonidentifiability. The capped binary construction establishes a sampling lower bound with an explicit low-information truncation. Exact feasible projection gives a robust upper bound against adaptive reports from identities fixed before the public codebook. Matching up to logarithms is limited to all conditions of Proposition 2. Neither arbitrary-budget matching nor general low-information optimality has been established. The fixed-sparsity decoder does not require the contamination fraction; the unknown-sparsity extension requires a known upper bound. The experiments support a conditional utility claim: JPRS beats the tested private global baselines at the six synthetic endpoints, but beats zero only in the Zipf setting. The Gowalla result is dominated by the public other columns and is weaker than some fixed public-structure references. Bounded attack searches complement the theory without certifying a worst-case optimum. Supplementary Section S8 gives the revised reproduction workflow.

Supplementary Materials: The following supporting information can be downloaded at: https://www.mdpi.com/article/10.3390/e1010000/s1: Supplementary Sections S1–S8, containing complete model details, proofs, additional experiments, and reproduction instructions; Reproducibility Package

S1, containing the source code, 30 frozen synthetic populations, configuration files, the immutable result CSV, generated figures and tables, checksums, and exact commands.

The supplementary file contains the following sections: Section S1: Three Privacy Adjacencies and Complete Model Details; Section S2: PLDP Channel Contraction Lemmas; Section S3: Contamination Identifiability Proofs; Section S4: Minimax Sampling Lower-Bound Proofs; Section S5: Complete Estimator Upper-Bound Proofs; Section S6: Unknown Sparsity with a Known Contamination Bound; Section S7: Additional Experiments and Parameter Settings; Section S8: Reproducibility Instructions.

Figure S1: Declared attack objectives and bounded search. Panel (a) uses $n = 16{,}000$, $p = 4096$, $s = 128$, $k = 8$, H1, nominal privacy 0.5, and contamination 0.1. Panel (c) averages the six synthetic families at nominal privacy 1. The budget check in (b) uses $n = 500$, $p = 256$, $s = 32$, $k = 4$, H0, nominal privacy 0.75, and contamination 0.1. All panels show five-seed means without dispersion. Panel (c) compares the final search loss with its displacement initialization; it does not certify global optimality.

Figure S2: Secondary five-seed diagnostics. The decreasing chance line in (c) prevents direct comparison of unadjusted support scores across domain sizes. Panel (d) changes both dictionary size and the corresponding sparsity budget. Panels (a) and (c) include pointwise 95% Student-t CIs for the mean; (b) and (d) show means without dispersion.

Table S1: All five sample-size points used in each clean JPRS slope fit (five seeds per point, $p = 4096$, $s = 128$, $k = 8$). Controlled and high-information sweeps use H1; the theory sweep uses H0. Optional group counts distinguish configuration identity; the global estimator uses all reports.

Table S2: Paired JPRS-minus-comparator loss differences (20 seeds), with pointwise 95 percent t intervals. Raw exact two-sided sign-test probabilities and Holm adjustments over all 30 tests are both reported; negative differences favor JPRS.

Table S3: Log–log slopes fitted over all five prespecified sample sizes for each seed. Pointwise 95% Student-t intervals summarize five seed-specific slopes; a fitted slope is descriptive, not a proof of a minimax rate.

Table S4: Same-global-sketch projection ablation. Entries are mean squared losses over 20 paired seeds per synthetic scenario. Group selection is held out of this comparison.

Table S5: Exploratory constant-reference check after inspecting target concentration (512 columns). Each estimator places the fixed constant c in each public other column and zero elsewhere. All five tested constants are shown; none is selected as a tuned method. Entries are deterministic sparse-target losses, outside the 30 primary tests.

Table S6: Sequential family-call time in seconds (mean and standard deviation over five seeds), repeated with contemporaneous environment recording. One BLAS thread, $n = 16{,}000$, H1, nominal privacy 1, no attack. Payload excludes public randomness and identity metadata.

Table S7: Successive clean JPRS log–log slopes in the nominal-privacy-2 H1 sweep. Each entry averages five seed-specific slopes; it is not the slope of the arithmetic mean losses.

The supplementary file cites the following references, which are also included in the bibliography of this article: [9–14,18,19,24,25,27,36,37].

Author Contributions: Conceptualization, J.O. and J.L.; methodology, J.O.; software, J.O. and J.L.; validation, J.L.; formal analysis, J.O.; investigation, J.O. and J.L.; resources, J.O.; data curation, J.L.; writing—original draft preparation, J.O.; writing—review and editing, J.O. and J.L.; visualization, J.L.; supervision, J.O.; project administration, J.O.; funding acquisition, J.O. All authors have read and agreed to the published version of the manuscript.

Funding: This research was funded by the Philosophy and Social Sciences Planning Project of Heyuan City, Guangdong Province (Key Project), grant number HYSK25Z13.

Institutional Review Board Statement: Not applicable.

Informed Consent Statement: Not applicable.

Data Availability Statement: The source code, 30 frozen synthetic populations, configuration files, raw experimental result CSV, plotting scripts, and checksums are provided in Reproducibility Package S1. MovieLens 1M, Foursquare NYC–Tokyo, and Gowalla are third-party datasets and are not

redistributed where their licenses do not clearly permit redistribution. Official source URLs, source hashes, and deterministic preprocessing instructions are included in Package S1 so that the six processed real-data arrays can be rebuilt from verified source archives.

Acknowledgments: ChatGPT (GPT-6, OpenAI) was used for language polishing and computational assistance.

Conflicts of Interest: The authors declare no conflicts of interest.

Abbreviations

The following abbreviations are used in this manuscript:

LDP	Local differential privacy
PLDP	Personalized local differential privacy
JPRS	Joint personalized relation sketch
HR	Hadamard response
OLH	Optimized local hashing
GRR	Generalized randomized response
P-RS	Padded relation sampling
DP	Differential privacy
TV	Total variation
CI	Confidence interval
NFE	Normalized Frobenius error
RAPPOR	Randomized Aggregatable Privacy-Preserving Ordinal Response
H0/H1/H2	Equal/moderate/wider public-budget profiles

References

1. Warner, S.L. Randomized Response: A Survey Technique for Eliminating Evasive Answer Bias. *J. Am. Stat. Assoc.* **1965**, *60*, 63–69. https://doi.org/10.1080/01621459.1965.10480775.
2. Kasiviswanathan, S.P.; Lee, H.K.; Nissim, K.; Raskhodnikova, S.; Smith, A. What Can We Learn Privately? *SIAM J. Comput.* **2011**, *40*, 793–826. https://doi.org/10.1137/090756090.
3. Wu, H.; Ran, R.; Peng, S.; Yang, M.; Guo, T. Mining frequent items from high-dimensional set-valued data under local differential privacy protection. *Expert Syst. Appl.* **2023**, *234*, 121105. https://doi.org/10.1016/j.eswa.2023.121105.
4. Shen, G.; Cai, M.; Huang, Z.; Yang, Y.; Guo, F.; Wei, L. LoHDP: Adaptive local differential privacy for high-dimensional data publishing. *Concurr. Comput. Pract. Exp.* **2024**, *36*, e8039. https://doi.org/10.1002/cpe.8039.
5. Ma, Y.; Jia, K.; Yang, H. Better Locally Private Sparse Estimation Given Multiple Samples Per User. In *Proceedings of the 41st International Conference on Machine Learning*; PMLR (Proceedings of Machine Learning Research): Cambridge, MA, USA, 2024; Volume 235, pp. 33746–33776. Available online: https://proceedings.mlr.press/v235/ma24c.html (accessed on 3 October 2026).
6. Cheng, L.; Lan, Q.; Yin, X.; Jia, S.; Sun, L. Adaptive utility optimization for personalized local differential privacy. *Expert Syst. Appl.* **2025**, *294*, 128750. https://doi.org/10.1016/j.eswa.2025.128750.
7. Wang, T.; Liang, J.; Wang, S.; Zhao, L.; Yang, T. Efficient and accurate graph statistics with adaptive personalized local differential privacy. *Neurocomputing* **2025**, *639*, 130224. https://doi.org/10.1016/j.neucom.2025.130224.
8. Li, M.; Berrett, T.B.; Yu, Y. On Robustness and Local Differential Privacy. *Ann. Stat.* **2023**, *51*, 717–737. https://doi.org/10.1214/23-AOS2267.
9. Chhor, J.; Sentenac, F. Robust Estimation of Discrete Distributions under Local Differential Privacy. In *Proceedings of the 34th International Conference on Algorithmic Learning Theory*; PMLR (Proceedings of Machine Learning Research): Cambridge, MA, USA, 2023; Volume 201, pp. 411–446. Available online: https://proceedings.mlr.press/v201/chhor23a.html (accessed on 3 October 2026).
10. Cao, X.; Jia, J.; Gong, N.Z. Data Poisoning Attacks to Local Differential Privacy Protocols. In *Proceedings of the 30th USENIX Security Symposium*; USENIX Association: Berkeley, CA, USA, 2021; pp. 947–964. Available online: https://www.usenix.org/conference/usenixsecurity21/presentation/cao-xiaoyu (accessed on 3 October 2026).
11. Wu, Y.; Cao, X.; Jia, J.; Gong, N.Z. Poisoning Attacks to Local Differential Privacy Protocols for Key-Value Data. In *Proceedings of the 31st USENIX Security Symposium*; USENIX Association: Berkeley, CA, USA, 2022; pp. 519–536. Available online: https://www.usenix.org/conference/usenixsecurity22/presentation/wu-yongji (accessed on 3 October 2026).
12. Li, X.; Li, N.; Sun, W.; Gong, N.Z.; Li, H. Fine-Grained Poisoning Attack to Local Differential Privacy Protocols for Mean and Variance Estimation. In *Proceedings of the 32nd USENIX Security Symposium*; USENIX Association: Berkeley, CA, USA, 2023;

pp. 1739–1756. Available online: https://www.usenix.org/conference/usenixsecurity23/presentation/li-xiaoguang (accessed on 3 October 2026).
13. Duchi, J.C.; Jordan, M.I.; Wainwright, M.J. Minimax Optimal Procedures for Locally Private Estimation. *J. Am. Stat. Assoc.* **2018**, *113*, 182–201. https://doi.org/10.1080/01621459.2017.1389735.
14. Kairouz, P.; Oh, S.; Viswanath, P. Extremal Mechanisms for Local Differential Privacy. *J. Mach. Learn. Res.* **2016**, *17*, 1–51. Available online: https://jmlr.org/papers/v17/15-135.html (accessed on 3 October 2026).
15. Asi, H.; Feldman, V.; Talwar, K. Optimal Algorithms for Mean Estimation under Local Differential Privacy. In *Proceedings of the 39th International Conference on Machine Learning*; PMLR (Proceedings of Machine Learning Research): Cambridge, MA, USA, 2022; Volume 162, pp. 1046–1056. Available online: https://proceedings.mlr.press/v162/asi22b.html (accessed on 3 October 2026).
16. Yang, M.; Guo, T.; Zhu, T.; Tjuawinata, I.; Zhao, J.; Lam, K.Y. Local differential privacy and its applications: A comprehensive survey. *Comput. Stand. Interfaces* **2024**, *89*, 103827. https://doi.org/10.1016/j.csi.2023.103827.
17. Erlingsson, Ú.; Pihur, V.; Korolova, A. RAPPOR: Randomized Aggregatable Privacy-Preserving Ordinal Response. In *Proceedings of the 2014 ACM SIGSAC Conference on Computer and Communications Security*; Association for Computing Machinery: New York, NY, USA, 2014; pp. 1054–1067. https://doi.org/10.1145/2660267.2660348.
18. Wang, T.; Blocki, J.; Li, N.; Jha, S. Locally Differentially Private Protocols for Frequency Estimation. In *Proceedings of the 26th USENIX Security Symposium*; USENIX Association: Berkeley, CA, USA, 2017; pp. 729–745. Available online: https://www.usenix.org/conference/usenixsecurity17/technical-sessions/presentation/wang-tianhao (accessed on 3 October 2026).
19. Acharya, J.; Sun, Z.; Zhang, H. Hadamard Response: Estimating Distributions Privately, Efficiently, and with Little Communication. In *Proceedings of the 22nd International Conference on Artificial Intelligence and Statistics*; PMLR (Proceedings of Machine Learning Research): Cambridge, MA, USA, 2019; Volume 89, pp. 1120–1129. Available online: https://proceedings.mlr.press/v89/acharya19a.html (accessed on 3 October 2026).
20. Cormode, G.; Maddock, S.; Maple, C. Frequency Estimation under Local Differential Privacy. *Proc. VLDB Endow.* **2021**, *14*, 2046–2058. https://doi.org/10.14778/3476249.3476261.
21. Zhang, M.; Lin, S.; Yin, L. Local differentially private frequency estimation based on learned sketches. *Inf. Sci.* **2023**, *649*, 119667. https://doi.org/10.1016/j.ins.2023.119667.
22. Charikar, M.; Chen, K.; Farach-Colton, M. Finding Frequent Items in Data Streams. *Theor. Comput. Sci.* **2004**, *312*, 3–15. https://doi.org/10.1016/S0304-3975(03)00400-6.
23. Donoho, D.L. Compressed Sensing. *IEEE Trans. Inf. Theory* **2006**, *52*, 1289–1306. https://doi.org/10.1109/TIT.2006.871582.
24. Wang, S.; Li, J.; Qian, Y.; Du, J.; Lin, W.; Yang, W. Hiding Numerical Vectors in Local Private and Shuffled Messages. In Proceedings of the Thirtieth International Joint Conference on Artificial Intelligence, Montreal, QC, Canada, 19–27 August 2021; pp. 3706–3712. https://doi.org/10.24963/ijcai.2021/510.
25. Zhou, M.; Wang, T.; Chan, T.H.H.; Fanti, G.; Shi, E. Locally Differentially Private Sparse Vector Aggregation. In *Proceedings of the 2022 IEEE Symposium on Security and Privacy*; IEEE: Piscataway, NJ, USA, 2022; pp. 422–439. https://doi.org/10.1109/SP46214.2022.9833635.
26. Kuang, X.; Zhu, Y.; Liu, R.; Zhang, S. Frequency estimation under relaxed input-discriminative local differential privacy. *Inf. Sci.* **2025**, *718*, 122351. https://doi.org/10.1016/j.ins.2025.122351.
27. Huber, P.J. Robust Estimation of a Location Parameter. *Ann. Math. Stat.* **1964**, *35*, 73–101. https://doi.org/10.1214/aoms/1177703732.
28. Lugosi, G.; Mendelson, S. Mean Estimation and Regression under Heavy-Tailed Distributions: A Survey. *Found. Comput. Math.* **2019**, *19*, 1145–1190. https://doi.org/10.1007/s10208-019-09427-x.
29. Yin, D.; Chen, Y.; Ramchandran, K.; Bartlett, P. Byzantine-Robust Distributed Learning: Towards Optimal Statistical Rates. In *Proceedings of the 35th International Conference on Machine Learning*; PMLR (Proceedings of Machine Learning Research): Cambridge, MA, USA, 2018; Volume 80, pp. 5650–5659. Available online: https://proceedings.mlr.press/v80/yin18a.html (accessed on 3 October 2026).
30. Zhu, H.; Ling, Q. Bridging Differential Privacy and Byzantine-Robustness via Model Aggregation. In Proceedings of the Thirty-First International Joint Conference on Artificial Intelligence, Vienna, Austria, 23–29 July 2022; pp. 2427–2433. https://doi.org/10.24963/ijcai.2022/337.
31. Qi, T.; Wang, H.; Huang, Y. Towards the Robustness of Differentially Private Federated Learning. In *Proceedings of the AAAI Conference on Artificial Intelligence*; AAAI Press: Washington, DC, USA, 2024; Volume 38; pp. 19911–19919. https://doi.org/10.1609/aaai.v38i18.29967.
32. Roy Chowdhury, A.; Imola, J.; Chaudhuri, K. Robust Locally Differentially Private Graph Analysis. In *Proceedings of the 20th ACM Asia Conference on Computer and Communications Security*; Association for Computing Machinery: New York, NY, USA, 2025; pp. 635–650. https://doi.org/10.1145/3708821.3736199.
33. Harper, F.M.; Konstan, J.A. The MovieLens Datasets: History and Context. *ACM Trans. Interact. Intell. Syst.* **2015**, *5*, 19:1–19:19. https://doi.org/10.1145/2827872.

34. Yang, D.; Zhang, D.; Zheng, V.W.; Yu, Z. Modeling User Activity Preference by Leveraging User Spatial Temporal Characteristics in LBSNs. *IEEE Trans. Syst. Man Cybern. Syst.* **2015**, *45*, 129–142. https://doi.org/10.1109/TSMC.2014.2327053.
35. Cho, E.; Myers, S.A.; Leskovec, J. Friendship and Mobility: User Movement in Location-Based Social Networks. In *Proceedings of the 17th ACM SIGKDD International Conference on Knowledge Discovery and Data Mining*; Association for Computing Machinery: New York, NY, USA, 2011; pp. 1082–1090. https://doi.org/10.1145/2020408.2020579.
36. Bassily, R.; Smith, A. Local, Private, Efficient Protocols for Succinct Histograms. In *Proceedings of the 47th Annual ACM Symposium on Theory of Computing*; Association for Computing Machinery: New York, NY, USA, 2015; pp. 127–135. https://doi.org/10.1145/2746539.2746632.
37. Shah, A.; Chen, W.-N.; Ballé, J.; Kairouz, P.; Theis, L. Optimal Compression of Locally Differentially Private Mechanisms. In *Proceedings of the 25th International Conference on Artificial Intelligence and Statistics*; PMLR (Proceedings of Machine Learning Research): Cambridge, MA, USA, 2022; Volume 151, pp. 7680–7723. Available online: https://proceedings.mlr.press/v151/shah22b.html (accessed on 3 October 2026).

Disclaimer/Publisher's Note: The statements, opinions and data contained in all publications are solely those of the individual author(s) and contributor(s) and not of MDPI and/or the editor(s). MDPI and/or the editor(s) disclaim responsibility for any injury to people or property resulting from any ideas, methods, instructions or products referred to in the content.
