# Supplementary material for: Information-Theoretic Limits and One-Bit Estimation of Sparse Relation Matrices Under Personalized Local Differential Privacy and Byzantine Contamination
Source: Entropy (Basel). 2026 Oct 5;28(10):1088. doi: 10.3390/e28101088 (PMC13703432; doi:10.3390/e28101088)
Supplement: Supplementary file 1 [file entropy-28-01088-s001.zip › entropy-4532751-supplementary/Package_S1.zip!/Package_S1/publication/proof_checks/entropy_sparse_relation_supplement.pdf]

Supplementary Materials: Information-Theoretic Limits and One-Bit Estimation of Sparse Relation Matrices Under Personalized Local Differential Privacy and Byzantine Contamination

Jia Ouyang [1] and Jinze Liu [2,*]

S1. Three Privacy Adjacencies and Complete Model Details

S1.1. Notation and Relational Observation Model

For a positive integer d, let $[d] = \{1, \dots, d\}$. The relation domain is

$$\Omega = [d_1] \times [d_2], \qquad p = |\Omega| = d_1 d_2.$$

For matrices A and B, let

$$\langle A, B\rangle = \sum_{u=1}^{d_1} \sum_{v=1}^{d_2} A_{uv} B_{uv}, \qquad \|A\|_F^2 = \langle A, A\rangle,$$
$$\|A\|_0 = |\{(u,v) : A_{uv} \neq 0\}|, \qquad \|A\|_1 = \sum_{u,v} |A_{uv}|.$$

Vectorization $\mathrm{vec}(A) \in \mathbb{R}^p$ is used only in the analysis. The local mechanism evaluates one public sign on each encoded relation pair; it does not factor a pair into row and column signs.

User i possesses a binary relation matrix $A_i \in \{0,1\}^{d_1 \times d_2}$, equivalently the active relation set

$$E_i = \{(u,v) \in \Omega : (A_i)_{uv} = 1\}.$$

Each user contributes at most k relations. The admissible user domain is

$$\mathcal{X}_k = \Big\{A \in \{0,1\}^{d_1 \times d_2} : \|A\|_0 \le k\Big\}.$$

All honest users have a common mean matrix

$$M = \mathbb{E}[A_i] \in [0,1]^{d_1 \times d_2},$$

but their full distributions need not be identical. The target is globally sparse. We work with

$$\mathcal{M}_{s,k} = \Big\{M \in [0,1]^{d_1 \times d_2} : \|M\|_0 \le s,\ \|M\|_1 \le k\Big\}, \tag{S1}$$

where $1 \le k \le p$ and $1 \le s \le p$. The ℓ_1 constraint is not an optional regularizer: it is forced by the per-user cardinality constraint because $\|M\|_1 = \mathbb{E}\|A_i\|_0 \le k$.

For $M \in \mathcal{M}_{s,k}$, define the admissible observation laws

$$\mathcal{P}_k(M) = \{P :\ P(\mathcal{X}_k) = 1,\ \mathbb{E}_{A \sim P}[A] = M\}. \tag{S2}$$

Conditional on the honest set, the honest observations are independent and $A_i \sim P_i$ for an arbitrary $P_i \in \mathcal{P}_k(M)$. Allowing different P_i's makes the upper bound distribution-free beyond boundedness, cardinality, and the common mean. The lower bound may select any convenient subfamily of $\mathcal{P}_k(M)$.

Proposition S1 (Realizability of the mean class)**.** *For $M \in [0,1]^{d_1 \times d_2}$, the class $\mathcal{P}_k(M)$ is nonempty if and only if $\|M\|_1 \le k$.*

Proof. If $A \in \mathcal{X}_k$ almost surely, then $\|\mathbb{E}A\|_1 = \sum_{u,v} \mathbb{E}A_{uv} = \mathbb{E}\|A\|_0 \le k$, proving necessity. For sufficiency, vectorize M as $m \in [0,1]^p$. The polytope

$$\mathcal{U}_{k,p} = \left\{ x \in [0,1]^p : \sum_{j=1}^{p} x_j \le k \right\}$$

is the independence polytope of the rank-k uniform matroid. Its constraint matrix is totally unimodular, hence every vertex is an incidence vector $x \in \{0,1\}^p$ with $\|x\|_0 \le k$. Therefore every $m \in \mathcal{U}_{k,p}$ is a convex combination $m = \sum_\ell \lambda_\ell x^{(\ell)}$ of such incidence vectors. Sampling $x^{(\ell)}$ with probability λ_ℓ and reshaping it into a matrix gives a law supported on $\mathcal{X}_k$ whose mean is M. □

Proposition S1 closes a modeling loophole that arises if one specifies only $\|M\|_0 \le s$ while simultaneously claiming that every user has at most k active relations. Without $\|M\|_1 \le k$, the proposed population mean may not correspond to any valid user-level data-generating process.

S1.2. Three Relational Privacy Units

For $A, A' \in \mathcal{X}_k$, write

$$\Delta(A, A') = \|A - A'\|_0 = |E(A) \triangle E(A')|,$$

where $\triangle$ denotes symmetric difference. We distinguish the following adjacency relations.

Definition S1 (Edge adjacency)**.** *$A \simeq_{\mathrm{e}} A'$ if $\Delta(A, A') = 1$. This protects the addition or removal of one relation.*

Definition S2 (Bounded relation-set adjacency)**.** *For $q \in [2k]$, $A \simeq_q A'$ if $1 \le \Delta(A, A') \le q$. This protects a simultaneous change affecting at most q relations.*

Definition S3 (User-level relation-set adjacency)**.** *$A \simeq_{\mathrm{u}} A'$ for every distinct $A, A' \in \mathcal{X}_k$. This protects replacement of the user's entire admissible relation set.*

The main results use Definition S3. Edge-level and bounded-set privacy are reported as corollaries because they protect strictly smaller changes. This choice is conservative: two k-relation sets can differ in $2k$ entries even though each is individually sparse.

Definition S4 (Personalized relational LDP)**.** *Let $Q_i(\cdot \mid A, u)$ be user i's local randomizer conditional on a public random seed $U = u$, with output space $\mathcal{Y}_i(u)$. For an adjacency relation $\simeq$, Q_i satisfies ε_i-personalized relational LDP if, for all $A \simeq A'$, all measurable $S \subseteq \mathcal{Y}_i(u)$, and every public seed u,*

$$Q_i(S \mid A, u) \le e^{\varepsilon_i} Q_i(S \mid A', u). \tag{S3}$$

A protocol satisfies personalized LDP if Eq. (S3) holds for every user with that user's announced budget ε_i.

Conditioning on the public seed in Definition S4 prevents shared randomness from silently weakening privacy. Personalized LDP means that the channels differ across users; it does not mean that the privacy budget may depend on the realized relation set. Throughout, ε_i is public, committed before data collection, and independent of A_i.

Proposition S2 (Privacy hierarchy)**.** *If Q_i satisfies ε_i-LDP under user-level adjacency, then it satisfies the same guarantee under q-set and edge adjacency. Conversely, an edge-private ε_i-LDP channel is at most $2k\varepsilon_i$-LDP under user-level adjacency.*

Proof. The first claim follows because $\simeq_{\mathrm{e}} \subseteq \simeq_q \subseteq \simeq_{\mathrm{u}}$. For the converse, take any $A, A' \in \mathcal{X}_k$. Remove the relations in $E(A) \setminus E(A')$ one at a time and then add the relations in $E(A') \setminus E(A)$ one at a time. Every intermediate matrix remains in $\mathcal{X}_k$, and the path has length $\Delta(A, A') \le \|A\|_0 + \|A'\|_0 \le 2k$. Multiplying the likelihood ratios along this path gives $Q_i(S \mid A, u) \le e^{2k\varepsilon_i} Q_i(S \mid A', u)$. □

Thus, presenting an edge-private mechanism as if it provided ε_i-user-level privacy can understate the worst-case privacy loss by a factor as large as $2k$. The joint sketch developed later is calibrated directly to Definition S3, avoiding this group-privacy loss.

S1.3. Noninteractive Personalized Protocol

A protocol is a tuple

$$\Pi = \left(U, \{Q_i\}_{i=1}^n, \widehat{M}\right).$$

The public random variable U, independent of all private data, specifies hashes, signs, and measurement assignments. Each honest user sends exactly one message

$$Z_i \sim Q_i(\,\cdot \mid A_i, U\,) \quad \text{with} \quad Z_i \in \mathcal{Y}_i(U).$$

There is no server-to-user message after data collection begins. The estimator receives U, the authenticated identities, the committed budget vector $\boldsymbol{\varepsilon} = (\varepsilon_1, \dots, \varepsilon_n)$, and the report vector $\mathbf{Z} = (Z_1, \dots, Z_n)$, and returns

$$\widehat{M} = \widehat{M}(U, \boldsymbol{\varepsilon}, \mathbf{Z}) \in [0,1]^{d_1 \times d_2}.$$

Conditional on $U = u$, user i's communication is

$$b_i(u) = \lceil \log_2 |\mathcal{Y}_i(u)| \rceil$$

bits. Public seeds and authenticated user identifiers are not charged to the per-user payload, following the public-coin communication convention used in succinct locally private protocols [1,2]. When the later mechanism assigns a public measurement index and transmits only a privatized sign, the private payload is one bit; any implementation-level header is reported separately.

S1.4. Response-Level Byzantine Contamination

Let

$$m_\alpha = \lfloor \alpha n \rfloor, \qquad \mathcal{B} \subseteq [n], \qquad |\mathcal{B}| \le m_\alpha,$$

be the Byzantine set and $\mathcal{H} = [n] \setminus \mathcal{B}$ the honest set. The server does not know either set. For $i \in \mathcal{H}$, Z_i is generated by the prescribed private channel. For $i \in \mathcal{B}$, the adversary may replace the message by any element of the valid output alphabet $\mathcal{Y}_i(U)$.

Formally, a response attack is a collection of stochastic maps

$$G_{\mathcal{B}} : \left(U, \varepsilon, \{Q_i\}_{i=1}^n, \{Z_j : j \in \mathcal{H}\}, \xi\right) \longmapsto \{Z_i^{\mathrm{adv}} \in \mathcal{Y}_i(U) : i \in \mathcal{B}\}, \tag{S4}$$

where ξ is arbitrary adversarial randomness. This definition permits coordination, knowledge of all public protocol components, knowledge of M, and observation of honest reports

before the forged reports are chosen. It therefore includes targeted support injection, sign-flip, high-budget-user corruption, and the output-poisoning attacks studied in [3–5].

Restricting forged reports to $\mathcal{Y}_i(U)$ is without loss for the proposed server: any out-of-domain message is first mapped to a fixed valid element by deterministic sanitization ($+1$ for the JPRS bit alphabet). This post-processing neither weakens honest-user privacy nor helps the adversary. The restriction is statistically important, however, because it prevents a defense from claiming robustness merely by rejecting malformed packets.

Response contamination is stronger than input contamination, in which a malicious user chooses a false $A_i \in \mathcal{X}_k$ but must still apply Q_i. The main theory uses response contamination; its upper bound also covers the smaller input-contamination class. Identities are authenticated, each identity has one committed budget and one report, and Sybil creation is excluded. Otherwise α would not bound the corrupted population.

The order of the suprema in Eq. (S5) also fixes the corrupted identity set after budgets are committed but before the fresh public pair-sign codebook is drawn. The forged report values may still depend on that codebook and on all honest reports, as Eq. (S4) permits. Allowing the adversary to choose which identities to corrupt *after* observing the fresh codebook is a stronger adaptive-compromise model and is outside the present scope.

S1.5. Minimax Risk and Identifiability

Let $\mathfrak{P}_k(M, \mathcal{H})$ be the collection of independent honest laws $\bigotimes_{i\in\mathcal{H}} P_i$ with $P_i \in \mathcal{P}_k(M)$, and let $\mathfrak{G}_\alpha$ be the response attacks in Eq. (S4). For a fixed protocol Π, define

$$\mathcal{R}(\Pi) = \sup_{M\in\mathcal{M}_{s,k}} \sup_{\substack{\mathcal{B}\subseteq[n] \\ |\mathcal{B}|\le m_\alpha}} \sup_{P_{\mathcal{H}}\in\mathfrak{P}_k(M,\mathcal{H})} \sup_{G_{\mathcal{B}}\in\mathfrak{G}_\alpha} \mathbb{E}\left[\left\|\widehat{M} - M\right\|_F^2\right]. \tag{S5}$$

The expectation includes honest data, local randomization, public randomness, and adversarial randomization. The loss is the unnormalized squared Frobenius error. The normalized Frobenius error (NFE) used in experiments divides complete-target squared loss by the complete target's squared norm; it does not replace the theoretical loss.

Let $\mathfrak{k}(\boldsymbol{\varepsilon}, \mathbf{b})$ be the class of single-round protocols satisfying user-level $\boldsymbol{\varepsilon}$-personalized LDP and per-user communication limits $\mathbf{b} = (b_1, \ldots, b_n)$. The communication-constrained minimax risk is

$$R^{\star}_{n,d_1,d_2,s,k}(\boldsymbol{\varepsilon}, \alpha, \mathbf{b}) = \inf_{\Pi\in\mathfrak{k}(\boldsymbol{\varepsilon},\mathbf{b})} \mathcal{R}(\Pi). \tag{S6}$$

When communication is unrestricted, $\mathbf{b}$ is omitted. The one-bit upper bound is compared with the unrestricted lower bound whenever possible; this is stronger than proving optimality only within the proposed channel family.

Definition S5 (Identifiability)**.** *Along a sequence of problem instances, $\mathcal{M}_{s,k}$ is identifiable if the unrestricted minimax risk in Eq.* (S6) *converges to zero. It is nonidentifiable if the risk is bounded below by a positive constant along some admissible sequence.*

Definition S5 separates finite-sample estimation from asymptotic recovery. In particular, a fixed positive response-contamination fraction generally creates an error floor; increasing n removes sampling noise but does not by itself reveal which of two contamination-compatible means generated the transcript. Section 4 of the main article formalizes this statement by a two-experiment contamination construction.

S1.6. Effective Private Information and Adversarial Leverage

Write $\beta_n = m_\alpha/n$ for the exact corrupted fraction and $\bar{k} = \min(k, s)$. Constants c, C, C_0 are positive and absolute, may change between displays, and do not depend on dimensions or budgets, unless dependence on α_0, C_γ is explicitly indicated.

For user i, define the LDP contraction coefficient

$$\gamma_i = \frac{e^{\varepsilon_i} - 1}{e^{\varepsilon_i} + 1} = \tanh(\varepsilon_i/2) \in [0, 1). \tag{S7}$$

For binary randomized response, a centered input signal is multiplied in expectation by γ_i; more generally, γ_i is the sharp total-variation contraction scale of an ε_i-LDP channel. In the strong-privacy regime $\varepsilon_i \le 1$, $\gamma_i^2 \asymp \varepsilon_i^2$ is also the relevant quadratic-information scale in private Le Cam, Fano, and Assouad bounds [6]. For unrestricted large budgets, however, a single scalar contraction coefficient does not describe the full information capacity of a high-alphabet channel; Section 4 of the main article therefore states the strong-privacy restriction whenever $\mathsf{I}_{\mathrm{eff}}^{(\alpha)}$ is used in a universal lower bound.

Because the adversary may occupy the most informative identities, define the effective information remaining after worst-case contamination

$$\mathsf{I}_{\mathrm{eff}}^{(\alpha)} = \min_{\substack{\mathcal{H} \subseteq [n] \\ |\mathcal{H}| = n - m_\alpha}} \sum_{i \in \mathcal{H}} \gamma_i^2. \tag{S8}$$

Equivalently, Eq. (S8) removes the m_α largest squared contraction coefficients. This is the worst-case information remaining for the sampling component of the risk.

Trimming information is not sufficient to describe response bias. We also define the effective adversarial leverage

$$\mathsf{L}_{\mathrm{eff}}^{(\alpha)} = \max_{\substack{\mathcal{B} \subseteq [n] \\ |\mathcal{B}| \le m_\alpha}} \frac{\sum_{i \in \mathcal{B}} \gamma_i}{\sum_{i \notin \mathcal{B}} \gamma_i^2}, \tag{S9}$$

with $\mathsf{L}_{\mathrm{eff}}^{(\alpha)} = 0$ if $m_\alpha = 0$, and $\mathsf{L}_{\mathrm{eff}}^{(\alpha)} = +\infty$ if $m_\alpha > 0$ and a denominator is zero. The numerator bounds the weighted displacement that valid one-bit Byzantine reports can create, while the denominator is the honest signal normalization. The fresh pair signs control this leverage uniformly for the global decoder, but no estimator can ignore privacy-dependent adversarial influence altogether.

Proposition S3 (Homogeneous-budget reduction). *If $\varepsilon_i = \varepsilon > 0$ for all i, with $\gamma = \tanh(\varepsilon/2)$, then*

$$\mathsf{I}_{\mathrm{eff}}^{(\alpha)} = (n - m_\alpha)\gamma^2, \qquad \mathsf{L}_{\mathrm{eff}}^{(\alpha)} = \frac{m_\alpha}{(n - m_\alpha)\gamma}.$$

For positive $\varepsilon \to 0$,

$$\mathsf{I}_{\mathrm{eff}}^{(\alpha)} = \frac{n(1-\beta_n)\varepsilon^2}{4}\{1 + O(\varepsilon^2)\}, \qquad \mathsf{L}_{\mathrm{eff}}^{(\alpha)} = \frac{2\beta_n}{(1-\beta_n)\varepsilon}\{1 + O(\varepsilon^2)\}.$$

For $\gamma = 0$, use the stated zero-denominator conventions instead.

Proof. All subsets of a fixed cardinality have the same sum in Eq. (S8). In Eq. (S9), the ratio increases with $|\mathcal{B}|$, so the maximum uses m_α users. Substituting the common γ yields the displayed identities. Finally, $\tanh(\varepsilon/2) = \varepsilon/2 + O(\varepsilon^3)$. □

Proposition S3 exposes the coupling that an additive "privacy error plus Byzantine error" expression misses. Under strong privacy, the sampling term loses a factor of order

ε^2, while the same valid forged bit has leverage of order $1/\varepsilon$ after signal normalization. For heterogeneous budgets, two populations with the same average budget can have radically different $\mathsf{I}_{\mathrm{eff}}^{(\alpha)}$: placing all large budgets on m_α identities permits the adversary to remove nearly all useful information. Neither $\min_i \varepsilon_i$ nor $n^{-1}\sum_i \varepsilon_i$ captures this effect.

S1.7. Standing Assumptions and Special Cases

The main results use the following assumptions.

A1. $1 \le k, s \le p = d_1 d_2$, and the target belongs to $\mathcal{M}_{s,k}$.

A2. Honest observations are independent, supported on $\mathcal{X}_k$, and have common mean M; no entrywise independence is assumed.

A3. The budgets $\varepsilon_i \ge 0$ are public, authenticated, and independent of private relation sets.

A4. The main robust regime has $0 \le \alpha < 1/2$. Results for $\alpha \ge 1/2$ are impossibility statements unless additional trusted structure is imposed.

A5. The adversary is response-level, coordinated, and computationally unbounded, but cannot forge identities or alter committed budgets. Its corrupted identity set is fixed before the fresh public pair-sign codebook; forged report values may be chosen after observing that codebook.

A6. The protocol is public-coin, noninteractive, and uses one report per identity.

A7. Matching private minimax rates are stated in the strong-privacy regime $\max_i \varepsilon_i \le 1$. Arbitrary finite budgets remain covered by the privacy and contamination definitions; large-budget and nonprivate limits are outside the sampling lower bound proved here. Matching additionally requires the information threshold, regularity, and corruption-count conditions of the main regular-budget proposition.

These assumptions yield several nested special cases. Setting $\alpha = 0$ gives personalized locally private sparse matrix estimation. Setting all $\varepsilon_i = \varepsilon$ gives homogeneous robust LDP. Taking $d_1 = 1$ reduces the target to a sparse frequency vector, while $p = s = 1$ reduces it to robust personalized scalar mean estimation. Letting $\varepsilon_i \to \infty$ removes privacy contraction and recovers a bounded Byzantine sparse-mean problem. These reductions are used as internal consistency checks whenever a theorem's stated privacy regime permits them; no nonprivate minimax theorem is claimed in this revision.

S2. PLDP Channel Contraction Lemmas

S2.1. Two Contraction Facts

For a Markov kernel Q and an input law P, write QP for the induced output law. For $\beta \in [0,1)$, define the response-contamination neighborhood

$$\mathsf{H}_\beta(R) = \{(1-\beta)R + \beta H : H \text{ is a probability measure on the same output space}\}. \tag{S10}$$

Lemma S1 (LDP contraction and neighborhood intersection)**.** *Let $0 \le \beta < 1$. Let Q be an ε-LDP channel under user-level adjacency and let $\gamma = \tanh(\varepsilon/2)$.*
(i) *For any input laws P_0, P_1 on $\mathcal{X}_k$,*

$$\mathrm{TV}(QP_0, QP_1) \le \gamma\, \mathrm{TV}(P_0, P_1). \tag{S11}$$

(ii) *For any probability measures R_0, R_1,*

$$\mathsf{H}_\beta(R_0) \cap \mathsf{H}_\beta(R_1) \neq \varnothing \quad \Longleftrightarrow \quad \mathrm{TV}(R_0, R_1) \le \frac{\beta}{1-\beta}. \tag{S12}$$

Proof. For part (i), fix two inputs x, x' and dominate $Q(\cdot \mid x)$ and $Q(\cdot \mid x')$ by a common measure, with densities q_x and $q_{x'}$. User-level LDP gives

$$e^{-\varepsilon} \le \frac{q_x(y)}{q_{x'}(y)} \le e^{\varepsilon}$$

whenever the ratio is defined. Hence

$$\frac{|q_x(y) - q_{x'}(y)|}{q_x(y) + q_{x'}(y)} \le \frac{e^{\varepsilon} - 1}{e^{\varepsilon} + 1} = \gamma.$$

Integration yields $\mathrm{TV}(Q(\cdot \mid x), Q(\cdot \mid x')) \le \gamma$. Thus the Dobrushin coefficient of Q is at most γ, and its variational characterization gives Eq. (S11). The coefficient is sharp and is attained by a binary staircase, equivalently randomized-response, channel [7].

For part (ii), the case $\beta = 0$ is exactly $R_0 = R_1$. For $0 < \beta < 1$, suppose a distribution R belongs to both neighborhoods. Then, for some H_0, H_1,

$$(1-\beta)(R_0 - R_1) = \beta(H_1 - H_0).$$

Taking total variation gives the necessity of Eq. (S12). Conversely, put $t = \mathrm{TV}(R_0, R_1)$. The least measure dominating both $(1-\beta)R_0$ and $(1-\beta)R_1$ has total mass $(1-\beta)(1+t)$. When $t \le \beta/(1-\beta)$, this mass is at most one. Complete it to a probability measure R by adding any nonnegative measure of the missing mass. Then

$$H_j = \frac{R - (1-\beta)R_j}{\beta}, \qquad j \in \{0, 1\},$$

is a probability measure and $R = (1-\beta)R_j + \beta H_j$. This proves sufficiency. □

Lemma S1 separates two roles of local privacy. Part (i) limits the distinguishability of honest experiments. Part (ii) states exactly how much valid response contamination is required to make two privatized experiments share the same observed distribution. Both statements hold for arbitrary output alphabets; see also [7–9].

S3. Contamination Identifiability Proofs

S3.1. A Response-Contamination Indistinguishability Radius

For $t \in [0, 1/2]$, define the budget-dependent corruption demand

$$\Phi_{\varepsilon}(t) = \sum_{i=1}^{n} \frac{\gamma_i t}{1 + \gamma_i t}. \tag{S13}$$

The response-contamination indistinguishability radius is

$$\varrho_{\mathrm{ind}}^{(\alpha)} = \sup\left\{ t \in [0, 1/2] : \Phi_{\varepsilon}(t) \le \frac{m_\alpha}{2} \right\}, \tag{S14}$$

with $\varrho_{\mathrm{ind}}^{(\alpha)} = 0$ when $m_\alpha = 0$. The factor $1/2$ in the corruption allowance is slack used to convert an independent-contamination construction into a hard cap of m_α Byzantine identities. Changing that factor changes constants, not the identifiability condition.

Proposition S4 (Scale of the indistinguishability radius)**.** *Let* $G_1 = \sum_{i=1}^{n} \gamma_i > 0$. *Then*

$$\min\left\{ \frac{1}{2}, \frac{m_\alpha}{2G_1} \right\} \le \varrho_{\mathrm{ind}}^{(\alpha)} \le \min\left\{ \frac{1}{2}, \frac{3m_\alpha}{4G_1} \right\}. \tag{S15}$$

For homogeneous positive budgets,

$$\varrho_{\mathrm{ind}}^{(\alpha)} = \min\left\{\frac{1}{2}, \frac{m_\alpha}{(2n - m_\alpha)\gamma}\right\}. \tag{S16}$$

Proof. For $0 \le t \le 1/2$ and $0 \le \gamma_i \le 1$,

$$\frac{2}{3}\gamma_i t \le \frac{\gamma_i t}{1+\gamma_i t} \le \gamma_i t.$$

Summing shows that $t = m_\alpha/(2G_1)$ is feasible whenever it is at most $1/2$, while every feasible t is at most $3m_\alpha/(4G_1)$. This proves Eq. (S15). If $\gamma_i = \gamma$, solving

$$\frac{n\gamma t}{1+\gamma t} \le \frac{m_\alpha}{2}$$

gives Eq. (S16). □

Theorem S1 (Response-contamination error floor)**.** *For every $m_\alpha \ge 0$, arbitrary finite personalized budgets, and unrestricted communication,*

$$R^\star_{n,d_1,d_2,s,k}(\varepsilon, \alpha) \ge \frac{\bar{k}(\varrho_{\mathrm{ind}}^{(\alpha)})^2}{16}\left(1 - 4e^{-m_\alpha/6}\right)_+. \tag{S17}$$

The factor is positive precisely for integer $m_\alpha \ge 9$; for $m_\alpha \le 8$ this bound is zero. Consequently, along any sequence with $m_\alpha \to \infty$, a positive lower limit of $\varrho_{\mathrm{ind}}^{(\alpha)}$ implies nonidentifiability.

Proof. If $m_\alpha \le 8$ or $\varrho_{\mathrm{ind}}^{(\alpha)} = 0$, the stated inequality is trivial. Otherwise fix an incidence matrix $A^\star$ supported on $\bar{k} = \min\{k, s\}$ relations and a number $t < \varrho_{\mathrm{ind}}^{(\alpha)}$. Consider the two valid capped submodels with correlated coordinates

$$M_0 = \left(\frac{1}{2} - \frac{t}{2}\right)A^\star, \qquad M_1 = \left(\frac{1}{2} + \frac{t}{2}\right)A^\star.$$

Under hypothesis h, an honest observation equals $A^\star$ with probability $1/2 + (-1)^{1-h}t/2$ and equals zero otherwise. Thus $\mathrm{TV}(P_0, P_1) = t$, and both means belong to $\mathcal{M}_{s,k}$.

Condition on a realization of the public seed. Let $R_{i,h} = Q_i P_h$ be user i's honest output law. By Lemma S1,

$$\mathrm{TV}(R_{i,0}, R_{i,1}) \le \gamma_i t.$$

Set

$$\beta_i(t) = \frac{\gamma_i t}{1+\gamma_i t}.$$

Then $\gamma_i t = \beta_i/(1-\beta_i)$, so Eq. (S12) supplies a common distribution $\widetilde{R}_i$ and attack distributions $H_{i,0}, H_{i,1}$ satisfying

$$\widetilde{R}_i = (1-\beta_i)R_{i,0} + \beta_i H_{i,0} = (1-\beta_i)R_{i,1} + \beta_i H_{i,1}. \tag{S18}$$

Because $\beta_i(t)$ depends only on public committed budgets, draw the independent indicators $\Xi_i \sim \mathrm{Bernoulli}(\beta_i)$ before the fresh public seed; hence the resulting corrupted identity set is independent of the codebook. Conditional on the subsequently drawn seed, if $\Xi_i = 0$, identity i reports honestly; if $\Xi_i = 1$, it reports from $H_{i,h}$. Before imposing the

hard cap, Eq. (S18) shows that the complete transcript has the identical product law $\bigotimes_i \widetilde{R}_i$ under $h = 0$ and $h = 1$. Moreover,

$$\mathbb{E}\sum_i \Xi_i = \Phi_\varepsilon(t) \le m_\alpha/2.$$

A multiplicative Chernoff bound therefore gives

$$\Pr\left(\sum_i \Xi_i > m_\alpha\right) \le e^{-m_\alpha/6}.$$

Condition the randomized attack on $\sum_i \Xi_i \le m_\alpha$. It is now a mixture over admissible fixed Byzantine sets, each of size at most m_α. Since a supremum over fixed sets dominates the risk averaged over such a mixture, this conditioning does not leave the minimax attack class. Each conditioned transcript law is within $2e^{-m_\alpha/6}$ in total variation of the common unconditioned law; hence the two conditioned laws are within $4e^{-m_\alpha/6}$ of each other.

Finally, $\|M_1 - M_0\|_F^2 = \bar{k}t^2$. Le Cam's two-point inequality for squared loss yields

$$\max_{h\in\{0,1\}} \mathbb{E}_h \left\|\widehat{M} - M_h\right\|_F^2 \ge \frac{\bar{k}t^2}{16}\left(1 - 4e^{-m_\alpha/6}\right)_+.$$

Letting $t \uparrow \varrho_{\mathrm{ind}}^{(\alpha)}$ proves the result. □

The proof uses a randomized prior over valid fixed Byzantine sets only as a lower-bound device. It does not enlarge the operational model: the maximum risk over deterministic sets is at least their prior average. The conditioning step is also why Eq. (S14) uses only half of the available corruption count.

Theorem S2 (Majority impossibility)**.** *If $2m_\alpha \ge n$, then for every privacy-budget vector and every protocol,*

$$R^\star_{n,d_1,d_2,s,k}(\varepsilon,\alpha) \ge \frac{\bar{k}}{4}. \tag{S19}$$

Proof. Take $M_0 = 0$ and $M_1 = A^\star$, where $A^\star$ has $\bar{k}$ active relations. Select sets $\mathcal{B}_0, \mathcal{B}_1$, each of cardinality at most m_α, whose union is $[n]$. No identity is honest in both experiments. In experiment zero, every identity in $\mathcal{B}_0$ simulates the report it would produce under experiment one whenever that identity is honest there. The adversary in experiment one performs the symmetric simulation. Identities corrupted in both experiments use a common arbitrary valid law. Conditional on every public seed, the two complete transcript laws are identical. For any estimate T, the parallelogram identity gives

$$\|T - M_0\|_F^2 + \|T - M_1\|_F^2 \ge \frac{1}{2}\|M_1 - M_0\|_F^2 = \frac{\bar{k}}{2}.$$

At least one of the two risks is therefore at least $\bar{k}/4$. □

S3.2. Necessary Conditions and Scope

Corollary S1 (General necessary conditions)**.** *For $\max_i \varepsilon_i \le 1$, with $B_{s,k}$ defined in Section S4,*

$$R^\star \ge c\left[B_{s,k}(\mathsf{I}_{\mathrm{eff}}^{(\alpha)}) \vee \bar{k}(\varrho_{\mathrm{ind}}^{(\alpha)})^2(1 - 4e^{-m_\alpha/6})_+\right]. \tag{S20}$$

Consequently, consistency requires

$$B_{s,k}(\mathsf{I}_{\mathrm{eff}}^{(\alpha)}) \to 0, \quad \bar{k}(\varrho_{\mathrm{ind}}^{(\alpha)})^2(1 - 4e^{-m_\alpha/6})_+ \to 0, \quad 2m_\alpha < n \text{ eventually.} \tag{S21}$$

Proof. Take the maximum of Theorems S3 and S1. The majority theorem excludes an infinite subsequence with $2m_\alpha \geq n$, since $\bar{k} \geq 1$. $\square$

Corollary S2 (Homogeneous fixed-structure consequence)**.** *For fixed s, k, homogeneous $0 < \varepsilon \leq 1$, and $m_\alpha \geq 9$ eventually, consistency requires*

$$(n - m_\alpha)\gamma^2 \to \infty, \qquad \beta_n/\gamma \to 0. \tag{S22}$$

When $\varepsilon \to 0$, these are

$$n(1-\beta_n)\varepsilon^2 \to \infty, \qquad \beta_n = o(\varepsilon). \tag{S23}$$

Proof. The first term of $B_{s,k}$ forces $\mathsf{I}_{\mathrm{eff}}^{(\alpha)} \to \infty$. For $m_\alpha \geq 9$, the finite-count factor is bounded below by $1 - 4e^{-9/6} > 0$. The homogeneous radius is comparable to $\min(1, \beta_n/\gamma)$, proving the second condition. Finally $\gamma \sim \varepsilon/2$. $\square$

When $m_\alpha = 0$, the contamination condition is absent. For $1 \leq m_\alpha \leq 8$, the displayed finite-count lower bound supplies no positive contamination floor; it must not be interpreted as proving zero contamination risk. For growing s, k, the general necessary conditions must be used. A condition $\mathsf{I}_{\mathrm{eff}}^{(\alpha)} \gg \bar{k}s$ does not follow without additional assumptions on the information regime.

S3.3. What the Lower Bounds Establish

The sampling theorem allows arbitrary noninteractive local channels and communication but requires $\max_i \varepsilon_i \leq 1$. It supplies a $\bar{k}s/\mathsf{I}_{\mathrm{eff}}^{(\alpha)}$ lower bound when $\mathsf{I}_{\mathrm{eff}}^{(\alpha)} \geq s^2/\bar{k}$. Below this threshold, the lower and upper bounds need not match. There is also a remaining support-search logarithm in the upper bound.

The radius $\varrho_{\mathrm{ind}}^{(\alpha)}$ is a protocol-independent lower-bound construction; $\mathsf{L}_{\mathrm{eff}}^{(\alpha)}$ is an upper-bound influence quantity. On bounded-ratio positive budget classes, it is $\min(1, \mathsf{L}_{\mathrm{eff}}^{(\alpha)})$, rather than the unbounded $\mathsf{L}_{\mathrm{eff}}^{(\alpha)}$, that is comparable to $\varrho_{\mathrm{ind}}^{(\alpha)}$. Section S5 gives explicit inequalities and the full conditions for matching. These restrictions do not affect the privacy guarantee itself.

S4. Minimax Sampling Lower-Bound Proofs

S4.1. A Capped Binary Block Construction

We give a direct construction on binary records. This avoids assuming that a signed-vector lower bound has the same low-information saturation as this mean class. Related local minimax and numerical-vector results include [6,10,11]; the argument below does not require extrapolating their homogeneous theorems.

Choose integers $q, r \geq 1$ with $q \leq k$ and $2qr \leq s$. Partition $2qr$ coordinates into q blocks, each containing r pairs. A record chooses exactly one coordinate independently in each block. For $v \in \{-1,1\}^{qr}$ and $0 < \delta \leq 1/2$, the block containing pair j chooses its coordinate (j, z), $z \in \{-1, 1\}$, with probability

$$P_v\{\text{block chooses } (j,z)\} = \frac{1 + \delta v_j z}{2r}.$$

Every record has exactly $q \leq k$ ones. Its mean has support $2qr \leq s$ and total mass q; all remaining coordinates are zero.

Let $\nu^{(j)}$ flip bit j, and write $Z_j = \pm 1$ when pair j is chosen with that sign, and $Z_j = 0$ otherwise. Under P_ν,

$$f_j = \frac{dP_{\nu^{(j)}}}{dP_\nu} - 1 = \frac{-2\delta\nu_j Z_j}{1+\delta\nu_j Z_j}, \qquad \mathbb{E}_\nu f_j = 0, \qquad \mathbb{E}_\nu f_j^2 = \frac{4\delta^2}{r(1-\delta^2)} =: v_\delta.$$

The scores are orthogonal: scores for distinct pairs in the same block have disjoint support, and scores in different blocks are independent and centered. Thus $\{f_j/\sqrt{v_\delta}\}$ is orthonormal in $L_2(P_\nu)$.

S4.2. Personalized Private Divergence Bound

Fix a public seed and identity i. On the finite input submodel use a common dominating output measure, write the channel density as $q_i(y|x)$, and set $m_\nu(y) = \mathbb{E}_\nu q_i(y|X)$. LDP implies

$$e^{-\varepsilon_i} \le q_i(y|x)/m_\nu(y) \le e^{\varepsilon_i}$$

where $m_\nu > 0$. Set $g_y(x) = q_i(y|x)/m_\nu(y) - 1$. The output likelihood difference in direction j is $\mathbb{E}_\nu(g_y f_j)$. Bessel's inequality gives

$$\begin{aligned}\sum_j \chi^2(Q_i P_{\nu^{(j)}} \| Q_i P_\nu) &= \int m_\nu(y) \sum_j \{\mathbb{E}_\nu(g_y f_j)\}^2 \, dy \\ &\le v_\delta \int m_\nu(y) \mathbb{E}_\nu g_y^2 \, dy \le v_\delta (e^{\varepsilon_i} - 1)^2.\end{aligned}$$

Since $\varepsilon_i \le 1$, $(e^{\varepsilon_i} - 1)^2 \le (e+1)^2 \gamma_i^2$. Also $\mathrm{KL}(P\|Q) \le \chi^2(P\|Q)$. Conditional tensorization over honest identities and then integration over the common public seed yield

$$\sum_{j=1}^{qr} \mathrm{KL}(\mathbb{P}^Z_{\nu^{(j)}} \| \mathbb{P}^Z_\nu) \le K\delta^2 I_H / r, \quad K = 16(e+1)^2/3, \quad I_H = \sum_{i \in H} \gamma_i^2. \tag{S24}$$

Here Z includes the public seed. The bound is uniform in the seed; shared public randomness therefore does not introduce dependence into the conditional tensorization.

S4.3. Assouad Loss and a Scalar Submodel

Decode ν_j from the sign of $\widehat{M}_{j,+} - \widehat{M}_{j,-}$, resolving a zero difference by a fixed sign. The true difference is $\delta\nu_j/r$. A wrong decoded bit costs at least $\delta^2/(2r^2)$ in the sum of the two coordinate errors, by Cauchy–Schwarz. The average neighboring total variation, averaged over vertices and coordinates, is at most

$$\overline{\mathrm{TV}} \le \sqrt{K\delta^2 I_H/(2qr^2)}$$

by Pinsker and Jensen. Pairing neighboring testing errors under the uniform prior on ν gives

$$\sup_\nu \mathbb{E}_\nu \|\widehat{M} - M_\nu\|_2^2 \ge \frac{q\delta^2}{4r}(1 - \overline{\mathrm{TV}}).$$

Take $\delta^2 = \min\{1/4, qr^2/(8KI_H)\}$, using $\delta^2 = 1/4$ when $I_H = 0$. The average variation is at most $1/4$, and hence

$$\sup_\nu \mathbb{E}_\nu \|\widehat{M} - M_\nu\|_2^2 \ge c \min\{q/r, q^2 r/I_H\}. \tag{S25}$$

For $s \ge 2$, choose $q = \min\{k, \lfloor s/2 \rfloor\}$ and $r = \lfloor s/(2q) \rfloor$. Then $q \ge \bar{k}/3$, $q \le \bar{k}$, and $s/4 \le qr \le s/2$. Consequently Eq. (S25) is at least $c \min\{\bar{k}^2/s, \bar{k}s/I_H\}$.

A separate two-point construction supplies the missing zero-information scale. Fix an incidence vector $A^\star$ with $b = \bar{k}$ ones and let $P_\pm$ choose $A^\star$ with probability $(1 \pm \delta)/2$, and zero otherwise. The squared mean separation is $b\delta^2$. Writing its one likelihood score as above gives $\mathbb{E}f^2 = 4\delta^2/(1-\delta^2)$; the same calculation bounds transcript KL by $K\delta^2 I_H$. Taking $\delta^2 = \min\{1/4, 1/(8KI_H)\}$ keeps total variation at most $1/4$. Le Cam's testing inequality yields $cb/(1+I_H)$. For $s = 1$ this also implies $c\min\{1, 1/I_H\}$, within an absolute constant.

S4.4. Trimmed Sampling Theorem

Theorem S3 (Trimmed private sampling lower bound). *For $p \ge s \ge 1$, $k \ge 1$, $\max_i \varepsilon_i \le 1$, and $0 \le \alpha < 1/2$, write $b = \bar{k} = \min(k, s)$. Then*

$$R^\star \ge cB_{s,k}(\mathsf{I}_{\mathrm{eff}}^{(\alpha)}), \qquad B_{s,k}(I) = \frac{b}{1+I} \vee \min\{b^2/s, bs/I\}, \quad bs/0 := +\infty.$$

In particular, $I \ge s^2/b$ gives the lower scale bs/I.

Proof. Choose the corrupted identities as those with the m_α largest γ_i. They emit a data-independent legal output law, which may depend on the public seed but not the hypothesis. This choice is fixed before the seed. The remaining identities have $I_H = \mathsf{I}_{\mathrm{eff}}^{(\alpha)}$. Apply the two constructions above. Both obey the cap and the common-mean model, so the supremum over admissible laws dominates both risks. □

S5. Complete Estimator Upper-Bound Proofs

S5.1. Pair-Safe Public Signs and the Local Channel

Let $R_i(e)$ be independent Rademacher signs across identities and $\min\{p, k+2s+1\}$-wise independent over encoded pairs for each identity. Fully independent signs are a stronger theoretical instantiation. The experiments simulate sign arrays with separately seeded pseudorandom streams; this reproducibility convention is not a proof of information-theoretic independence for a finite seed. Public codebooks and private response coins must be independently generated in an actual protocol, and the simulation master seed must not be published as a deployment seed. For $m \ge 1$, define

$$a_m = m^{-1}\mathbb{E}\left|\sum_{j=1}^{m} \xi_j\right|, \quad \kappa_k = \sqrt{k}a_k, \quad c_k(m) = a_k/a_m, \quad c_k(0) = 0.$$

The variables ξ_j are independent Rademacher signs. Use $\mathrm{sgn}_0(0) = 0$ and the ordinary sign otherwise. An independent private fair tie coin followed by the bit randomizer has the same marginal output law; the tie coin is not included in the public seed. This convention makes the displayed channel a probability conditional only on the record and public signs. For a nonempty record E_i of size m, set

$$\theta_i(E_i) = c_k(m)\,\mathrm{sgn}_0\!\left(\sum_{e \in E_i} R_i(e)\right), \qquad \Pr(Y_i = y \mid E_i, R_i) = \frac{1 + y\gamma_i\theta_i(E_i)}{2},$$

and put $\theta_i(\varnothing) = 0$.

Lemma S2 (Privacy and exact set moment). *The JPRS channel is user-level ε_i-LDP and, for every honest identity and relation e,*

$$\mathbb{E}\{Y_i R_i(e)\} = \gamma_i \kappa_k M_e/\sqrt{k}. \tag{S26}$$

Proof. For every admissible set, $|\theta_i(A)| \leq 1$, so both output probabilities lie in $[(1-\gamma_i)/2, (1+\gamma_i)/2]$. Every likelihood ratio is bounded by $(1+\gamma_i)/(1-\gamma_i) = e^{\varepsilon_i}$, uniformly in the public seed. For a nonempty set of size m, exchangeability gives

$$\mathbb{E}_R\left[\operatorname{sgn}_0\left(\sum_{e' \in E_i} R_i(e')\right) R_i(e) \mid e \in E_i\right] = a_m.$$

Multiplication by $c_k(m)$ makes this value $\kappa_k/\sqrt{k}$, independent of m. It is zero when $e \notin E_i$. Finally $\mathbb{E}(Y_i \mid A_i, R_i) = \gamma_i \theta_i(A_i)$ and $\Pr(e \in E_i) = M_e$, proving Eq. (S26). □

Calibration and constants.

The random-walk identity

$$a_m = 2^{-(m-1)} \binom{m-1}{\lfloor (m-1)/2 \rfloor}$$

follows by pairing symmetric binomial terms in $\mathbb{E}|\sum_{j=1}^m \xi_j|$. For $\ell \geq 1$, $a_{2\ell} = a_{2\ell+1}$ and $a_{2\ell}/a_{2\ell-1} = (2\ell-1)/(2\ell)$, proving monotonicity. Thus the former minimum $\min_{m \leq k} \sqrt{k} a_m$ equals $\sqrt{k} a_k$. The sharp lower Khintchine bound and Cauchy–Schwarz give $\sqrt{k/2} \leq \mathbb{E}|\sum_{j=1}^k \xi_j| \leq \sqrt{k}$, hence $1/\sqrt{2} \leq \kappa_k \leq 1$. For an honest set J, $D_J > 0$, independence across identities gives

$$\operatorname{Var}\left(\frac{1}{a_k D_J} \sum_{i \in J} \gamma_i Y_i R_i(e)\right) \leq \frac{1}{a_k^2 D_J} = \frac{k}{\kappa_k^2 D_J} \leq \frac{2k}{D_J}.$$

For unbiased linear weights w_i, the constraint is $a_k \sum_{i \in J} w_i \gamma_i = 1$. Cauchy–Schwarz implies $\sum_i w_i^2 \geq 1/(a_k^2 D_J)$, with equality for $w_i = \gamma_i/(a_k D_J)$. This optimizes the uniform variance bound $\sum_i w_i^2$; it does not assert optimal actual variance for every possible law. At $k = 8$, $1/\kappa_k^2 = 2048/1225$, not 2. An empty record has zero mean score, releases a fair bit, and remains in D_J or the global public denominator. The guarantee concerns one report; repeated participation requires privacy composition across releases.

S5.2. Sparse Projection and Clean Global Concentration

Let $\mathcal{H}_q(x)$ retain the $\min(q, p)$ largest coordinates of x in absolute value, breaking ties by increasing coordinate index. Define $\mathcal{P}_{s,k}$ as an exact Euclidean minimizer over $\mathcal{M}_{s,k}$. The following lemma specifies a canonical minimizer and justifies the repaired implementation.

Lemma S3 (Sparse box-and-mass projection)**.** *Let $v \in \mathbb{R}^p$, $s \in \{0, \ldots, p\}$, and $k \geq 0$. Let S consist of the first s positive coordinates in descending raw value, breaking ties by increasing index (use all positive coordinates if there are fewer). An exact projection onto $\{x \in [0,1]^p : \|x\|_0 \leq s, \sum_j x_j \leq k\}$ is zero off S, and on S equals*

$$x_j = \min\{1, \max\{0, v_j - \lambda\}\}.$$

If $k = 0$, return zero. Otherwise use $\lambda = 0$ when the selected clipped mass is at most k; if it exceeds k, choose $\lambda \geq 0$ with $\sum_{j \in S} x_j = k$.

Proof. A nonpositive coordinate of v never needs positive fitted mass: setting it to zero weakly reduces loss and preserves feasibility. If a feasible vector has $x_j = a > 0$, $x_i = 0$, and $v_i \geq v_j$, moving mass a from j to i changes its squared distance by $2a(v_j - v_i) \leq 0$. Repeated exchanges yield a minimizer supported on S, with the stated canonical tie rule. On that fixed support the objective is strictly convex. Its box-constrained Lagrange optimality conditions give $x_j = \min(1, \max(0, v_j - \lambda))$ and $\lambda(\sum_j x_j - k) = 0$. The selected mass is a

continuous nonincreasing function of λ, ranging from its value at zero to zero, so an active threshold exists. The fitted vector is unique on the selected support, even if a multiplier is nonunique on a plateau. □

The repaired numerical routine uses this formula with a centered multiplier and bisection. It never clips v before computing the active threshold. For example, $v = (2, 0.9)$, $s = 2, k = 1$ has projection $(1, 0)$, with squared distance 1.81. Pre-clipping and then applying an ordinary simplex projection gives $(0.55, 0.45)$, whose squared distance to the original v is 2.305. Feasibility alone would miss this failure.

The numerical implementation is checked against enumeration of all small constraint faces and independent constrained optimization. These finite tests do not replace the mathematical lemma. More generally, if a feasible numerical output has objective gap at most τ relative to the exact minimum, the next proof gives $\|\widehat{M} - M\|_2 \le 2\|\mathcal{H}_{2s}(W)\|_2 + \sqrt{\tau}$. The stated statistical theorem uses $\tau = 0$.

Lemma S4 (Feasible projection inequality). *For $M \in \mathcal{M}_{s,k}$, $V = M + W$, and any exact Euclidean projection $\widehat{M} \in \mathcal{P}_{s,k}(V)$,*

$$\|\widehat{M} - M\|_2 \le 2\|\mathcal{H}_{2s}(W)\|_2.$$

Proof. Projection optimality gives $\|V - \widehat{M}\|_2^2 \le \|V - M\|_2^2$. After expansion, $\|\widehat{M} - M\|_2^2 \le 2\langle \widehat{M} - M, W\rangle$. The difference is supported on at most $2s$ coordinates, so Cauchy–Schwarz proves the claim. □

Fix an admissible Byzantine set B, put $H = [n] \setminus B$, and define $I_H = \sum_{i \in H} \gamma_i^2$, $L_B = \sum_{i \in B} \gamma_i$, and $D = \sum_i \gamma_i^2$. Introduce independent honest ghost records and bits for identities in B, and let

$$V^0(e) = \frac{\sqrt{k}}{\kappa_k D} \sum_{i=1}^{n} \gamma_i Y_i^0 R_i(e).$$

Lemma S2 gives $\mathbb{E} V^0 = M$; importantly, the observable denominator D is the same before and after ghost replacement.

Lemma S5 (Uniform clean sparse deviation). *For $1 \le q \le \min(2s, p)$, let $T_q(\delta) = q \log(9ep/q) + \log(8/\delta)$. If $D > 0$, then with probability at least $1 - \delta/4$,*

$$\|\mathcal{H}_q(V^0 - M)\|_2^2 \le CkT_q(\delta)/D.$$

Proof. For a fixed support S, $|S| = q$, and a unit $u \in \mathbb{R}^S$, the centered projection $\langle u, V^0 - M\rangle$ is a sum over identities. The union of S and an honest active set has at most $q + k$ pairs, so the stated finite-wise independence makes its joint sign law identical to fully independent Rademachers for this calculation. For identity i, multiplication by $Y_i^0 \in \{-1, 1\}$ does not change the absolute value of $\langle u, R_{i,S}\rangle$. The Rademacher Khintchine inequality therefore gives

$$\left\| \frac{\sqrt{k}\gamma_i}{\kappa_k D} Y_i^0 \langle u, R_{i,S}\rangle \right\|_{\psi_2} \le \frac{C\sqrt{k}\gamma_i}{D}.$$

Centering changes the Orlicz norm by at most a universal factor. Independence across identities and $\sum_i \gamma_i^2 = D$ imply

$$\Pr\{|\langle u, V^0 - M\rangle| > t\} \le 2\exp\{-cDt^2/k\}.$$

Use a 1/4-net of the unit sphere with at most 9^q points and $\binom{p}{q} \le (ep/q)^q$ supports. A union bound and the standard net approximation multiply the radius by at most two. Maximizing over supports is exactly the top-q norm. Notice that no sample-size regularity is needed for this clean term; Eq. (S27) is used only to absorb the mixed valid-output attack term. □

S5.3. A Uniform Valid-Output Attack Bound

Lemma S6 (Signed-output operator event)**.** *For $1 \le q \le \min(2s, p)$, if B is fixed before the pair signs, then with probability at least $1-\delta/4$, simultaneously for every $|S| \le q$ and every adaptive coefficient vector $b \in [-2,2]^B$,*

$$\left\| \sum_{i\in B} \gamma_i b_i R_{i,S} \right\|_2 \le C\left\{ L_B + \sqrt{\gamma_{\max} L_B T_q(\delta)} \right\}.$$

Proof. For a fixed support S, $|S| = q$, and unit vector u on S,

$$\sup_{b\in[-2,2]^B} \left| \left\langle u, \sum_{i\in B} \gamma_i b_i R_{i,S} \right\rangle \right| \le 2 \sum_{i\in B} \gamma_i |\langle u, R_{i,S}\rangle|.$$

The expectation of each absolute Rademacher sum is at most one. Its centered version has subgaussian norm bounded by an absolute constant, since $\|u\|_2 = 1$. Independence across identities implies, with probability at least $1 - 2e^{-t}$,

$$\sum_{i\in B} \gamma_i |\langle u, R_{i,S}\rangle| \le L_B + C\sqrt{t \sum_{i\in B} \gamma_i^2} \le L_B + C\sqrt{t\gamma_{\max} L_B}.$$

Take a 1/4-net of size at most 9^q for each support and union bound over at most $(ep/q)^q$ supports. Taking $t = q\log(9ep/q) + \log(8/\delta)$ gives failure probability at most $\delta/4$. For each vector the net approximation costs at most 4/3; taking the supremum over b preserves this bound. Supports smaller than q are included by padding. This proves the displayed inequality uniformly in b, even if b is selected after observing all signs and honest reports. The case $B = \varnothing$ is identically zero. No union bound over attack strategies is needed. □

S5.4. Proof of the Global Recovery Theorem

Put $q_\star = \min(2s, p)$. For the remainder of Section S5 assume

$$k \le s, \qquad \mathsf{I}_{\mathrm{eff}}^{(\alpha)} > 0, \qquad \mathsf{I}_{\mathrm{eff}}^{(\alpha)} \ge C_0 \gamma_{\max}^2 T_{q_\star}(\delta), \tag{S27}$$

and that the corrupted identity set is fixed before the fresh pair signs.

Theorem S4 (Global JPRS upper bound)**.** *Under Eq. (S27), with probability at least $1-\delta$,*

$$\|\widehat{M} - M\|_2^2 \le Ck\left\{ \frac{T_{q_\star}(\delta)}{\mathsf{I}_{\mathrm{eff}}^{(\alpha)}} + (\mathsf{L}_{\mathrm{eff}}^{(\alpha)})^2 \right\}.$$

Proof. Write $b_i = \overline{Y}_i - Y_i^0 \in [-2,2]$ on B. Lemma S6, with $q = q_\star$, gives

$$\|\mathcal{H}_{2s}(V - V^0)\|_2 \le \frac{C\sqrt{k}}{D}\{L_B + \sqrt{\gamma_{\max} L_B T_{q_\star}}\}.$$

Let $a = L_B/D$ and $b = \gamma_{\max} T_{q_\star}/D$. Squaring the right-hand side and using $ab \le (a^2 + b^2)/2$ gives $Ck(a^2 + b^2)$. Since $D \ge I_H \ge \mathsf{I}_{\mathrm{eff}}^{(\alpha)}$, $a \le L_B/I_H \le \mathsf{L}_{\mathrm{eff}}^{(\alpha)}$. Moreover, Eq. (S27) implies

$$b^2 = \frac{\gamma_{\max}^2 T_{q_\star}}{D} \frac{T_{q_\star}}{D} \le C \frac{T_{q_\star}}{D} \le C \frac{T_{q_\star}}{\mathsf{I}_{\mathrm{eff}}^{(\alpha)}}.$$

Lemma S5 controls $V^0 - M$ at the same sampling scale. The triangle inequality and Lemma S4 prove the claim. The clean and attack events each fail with probability at most $\delta/4$, so their union fails with probability at most $\delta/2 \le \delta$. No union over attack strategies is taken because the operator event is already uniform. □

Corollary S3 (Expected risk)**.** *For $n \ge 2$, assuming Eq. (S27) with $\delta = n^{-2}$,*

$$\mathcal{R}(\Pi_{\mathrm{JPRS}}) \le C \left[\bar{k} \wedge k \left\{ \frac{s \log(9epn)}{\mathsf{I}_{\mathrm{eff}}^{(\alpha)}} + (\mathsf{L}_{\mathrm{eff}}^{(\alpha)})^2 \right\} \right] + 2\bar{k}/n^2.$$

Proof. For every outcome, feasibility and nonnegativity give $\|\widehat{M} - M\|_2^2 \le \|\widehat{M}\|_2^2 + \|M\|_2^2 \le 2\bar{k}$. On the success event, take the minimum of this deterministic bound and the theorem bound. On the failure event use $2\bar{k}$; its probability is at most n^{-2}. Since $T_{q_\star}(n^{-2}) \le Cs \log(9epn)$, integration proves the result for this same estimator. No switch to a different estimator is required. □

S5.5. Regular Budgets and Optional Grouped Decoders

Let $g = \gamma_{\min} > 0$, $\gamma_i \in [g, C_\gamma g]$, and $\alpha \le \alpha_0 < 1/2$. For $\beta_n = m_\alpha/n > 0$, explicit bounds are

$$\frac{\beta_n}{(1-\beta_n) C_\gamma^2 g} \le \mathsf{L}_{\mathrm{eff}}^{(\alpha)} \le \frac{C_\gamma \beta_n}{(1-\beta_n) g}.$$

The lower bound uses any set of size m_α; the upper bound applies to every smaller set as well. With $ng \le G_1 \le nC_\gamma g$, Eq. (S15) gives

$$\min\{1/2, \beta_n/(2C_\gamma g)\} \le \varrho_{\mathrm{ind}}^{(\alpha)} \le \min\{1/2, 3\beta_n/(4g)\}.$$

Thus $\mathsf{L}_{\mathrm{eff}}^{(\alpha)} \asymp \beta_n/g$ and $\min(1, \mathsf{L}_{\mathrm{eff}}^{(\alpha)}) \asymp \varrho_{\mathrm{ind}}^{(\alpha)} \asymp \min(1, \beta_n/g)$, with constants only depending on α_0, C_γ. When $m_\alpha = 0$, all these quantities are zero by convention.

For the matching statement, additionally assume $k \le s$, $\max_i \varepsilon_i \le 1$, $\mathsf{I}_{\mathrm{eff}}^{(\alpha)} \ge s^2/k$, and regularity at $\delta = n^{-2}$. The sampling lower bound then gives $cks/\mathsf{I}_{\mathrm{eff}}^{(\alpha)}$. If $m_\alpha \ge 9$, the contamination bound gives $c_{\alpha_0, C_\gamma} k \min\{1, (\mathsf{L}_{\mathrm{eff}}^{(\alpha)})^2\}$; if $m_\alpha = 0$, this term is absent. These terms bound the expected upper risk within a factor $C_{\alpha_0, C_\gamma} \log(epn)$: use $\min\{1, a + L^2\} \le a + \min\{1, L^2\}$ and $\mathsf{I}_{\mathrm{eff}}^{(\alpha)} \le n$ to absorb k/n^2 into $ks/\mathsf{I}_{\mathrm{eff}}^{(\alpha)}$. For $1 \le m_\alpha \le 8$, or below the information threshold, this argument does not establish matching.

For completeness, let public groups produce $V_1, \ldots, V_G$ and define $d_q(x, y) = \|\mathcal{H}_q(x - y)\|_2$. If a strict majority lies in a d_{2s}-ball of radius r around M, the candidate minimizing median pairwise distance is within $3r$ before projection and within $6r$ after Lemma S4. Averaging candidates inside its median radius gives the analogous $10r$ bound. These deterministic statements justify the medoid and pooling controls, but the stochastic model above puts the *global* sketch in the required ball directly; grouping would only replace D by group-level information and add variance.

S6. Unknown Sparsity with a Known Contamination Bound

The fixed-sparsity decoder needs s, k, budgets, and public signs, but not α. The adaptive extension below requires an additional public bound $\bar{\alpha} \geq \alpha$, with $\bar{m} = \lfloor \bar{\alpha} n \rfloor < n/2$. Define $\bar{I} = \mathsf{I}_{\mathrm{eff}}^{(\bar{\alpha})} > 0$ and $\bar{L} = \mathsf{L}_{\mathrm{eff}}^{(\bar{\alpha})}$ using the committed budgets. An oracle rule using the unknown true contamination is not an implementable parameter-free decoder.

Use fully independent public signs, and let $\mathcal{T} = \{2^j : j \geq 0,\ 2^j < p\} \cup \{p\}$. For $t \in \mathcal{T}$, put $q_t = \min(2t, p)$, $M^{(t)} \in \mathcal{P}_{t,k}(M)$, $a_t = \|M - M^{(t)}\|_2$, and $\widehat{M}_t = \mathcal{P}_{t,k}(V)$. This section permits any mean $M \in [0,1]^p$ with $\|M\|_1 \leq k$, including approximately sparse means. Choose an absolute constant C large enough for the concentration inequalities of Section S5, and define

$$r_t = C\sqrt{k\left\{\frac{q_t \log(9ep/q_t) + \log(8|\mathcal{T}|/\delta)}{\bar{I}} + \bar{L}^2\right\}}.$$

Assume scale-uniform regularity $\bar{I} \geq C_0 \gamma_{\max}^2 T_{q_t}(\delta/|\mathcal{T}|)$ for every $t \in \mathcal{T}$. Select the smallest t such that

$$\|\widehat{M}_t - \widehat{M}_u\|_2 \leq 4(r_t + r_u) \quad \text{for every } u \in \mathcal{T} \text{ with } u \geq t.$$

The final scale p always passes, so selection is well-defined. The constant is a conservative theoretical constant, not an estimated function of the unknown contamination. Numerical calibration and implementation of this optional extension are not claimed in the present experiments.

Proposition S5 (Dyadic sparsity adaptation)**.** *Under the above assumptions and the fixed-before-codebook attack model, with probability at least* $1 - \delta$*,*

$$\|\widehat{M}_{\hat{t}} - M\|_2^2 \leq C' \inf_{t \in \mathcal{T}} \{a_t^2 + r_t^2\}.$$

The entire family and selection use the same private reports, so this post-processing incurs no additional privacy or private communication cost.

Proof. The clean and attack concentration arguments of Section S5 hold without a sparse mean when public signs are fully independent. Allocate failure probability $\delta/|\mathcal{T}|$ to each scale. Since the actual corrupted set has size at most $\bar{m}$, the same bounds use $\bar{I}, \bar{L}$. On their common event, writing $W = V - M$, choose C so that $2\|\mathcal{H}_{q_t}(W)\|_2 \leq r_t$ for all t. Projection optimality against $M^{(t)}$ gives, for $d_t = \widehat{M}_t - M^{(t)}$,

$$\|d_t\|_2^2 \leq 2\langle d_t, W + M - M^{(t)}\rangle \leq 2\|d_t\|_2\{\|\mathcal{H}_{q_t}(W)\|_2 + a_t\}.$$

Therefore $e_t := \|\widehat{M}_t - M\|_2 \leq r_t + 3a_t$. The radii are nondecreasing and the approximation errors are nonincreasing. Let $t_\star = \min\{t : a_t \leq r_t\}$, which exists since $a_p = 0$. For $u \geq t_\star$, $e_u \leq 4r_u$, so $t_\star$ passes all comparisons. Minimality implies $\hat{t} \leq t_\star$, whence

$$e_{\hat{t}} \leq 4(r_{\hat{t}} + r_{t_\star}) + e_{t_\star} \leq 12 r_{t_\star}.$$

For adjacent scales, $r_{t_{j+1}}^2 \leq 2r_{t_j}^2$, since $q \log(9ep/q)$ is increasing on $[1, p]$, and doubling q increases this function by at most a factor two. If $t < t_\star$, and v is the predecessor of $t_\star$, then $a_t \geq a_v > r_v \geq r_{t_\star}/\sqrt{2}$. If $t \geq t_\star$, $r_t \geq r_{t_\star}$. Thus $r_{t_\star}^2 \leq 2\inf_t(a_t^2 + r_t^2)$, including the case $t_\star = 1$. Combining the bounds proves the proposition. □

For an exactly s-sparse mean, the first dyadic scale at least s is at most $2s$; its bias is zero. The additional union-bound term in the radius is $\log|\mathcal{T}| = O(\log\log(p+1))$. Scale-uniform regularity may be substantially stronger than the fixed-sparsity assumption and is an explicit cost of this extension.

S7. Additional Experiments and Parameter Settings

All figures and numerical tables below are regenerated from the corrected audited implementation. A separate sequential timing audit records its computational environment. The main article defines the targets, tie-averaged support metric, zero reference, and primary family of 30 comparisons.

S7.1. Implemented Comparison Channels

Every P-RS baseline samples one label J_i from the k padded record positions, including a public dummy label outside the p reconstructed relations. Consequently $\Pr(J_i = j \mid A_i) = A_i(j)/k$ for a real relation j. Each resulting channel protects replacement of the entire padded set. All global reconstructions receive the same feasible support/box/mass projection.

For the public-coin HR variant, let H be a Hadamard matrix of order $K = 2^{\lceil \log_2(p+1) \rceil}$. Identity i receives a uniform public row r_i and releases $Y_i = H_{r_i,J_i}$ with probability $(1+\gamma_i)/2$, otherwise its negative. Orthogonality gives $\mathbb{E}[Y_i H_{r_i,j}] = \gamma_i M_j/k$. The raw estimate is $k \sum_i \gamma_i Y_i H_{r_i,j} / \sum_i \gamma_i^2$. This shared-randomness variant uses one private bit and a direct scan; it does not inherit the original HR protocol's private-coin payload or fast-decoding benchmark[12].

For GRR on $q = p+1$ labels, write $r_i = (e^{\varepsilon_i} + q - 1)^{-1}$ and $a_i = (e^{\varepsilon_i} - 1)/(e^{\varepsilon_i} + q - 1)$. The sampled label is reported with probability $e^{\varepsilon_i} r_i$ and every alternative with probability r_i. Its raw estimate is $k \sum_i a_i(\mathbf{1}\{Y_i = j\} - r_i)/\sum_i a_i^2$. For OLH[13], choose $g_i = \max\{2, \mathrm{round}(e^{\varepsilon_i} + 1)\}$ (nearest-even rounding), apply the public hash defined below, and perform GRR on its g_i buckets. With $a_i = (g_i - 1)(e^{\varepsilon_i} - 1)/[g_i(e^{\varepsilon_i} + g_i - 1)]$, the raw estimate is $k \sum_i a_i(\mathbf{1}\{h_i(j) = Y_i\} - 1/g_i)/\sum_i a_i^2$. Zero information returns zero. These mechanism-specific gains describe normalization; the theorem-regime audit separately uses the common $\tanh(\varepsilon_i/2)$ channel amplitudes. Payload counts exclude public rows, hashes, authenticated identity metadata, and public randomness distribution.

The optional Medoid and Pooled outputs use public random groups and the same reports as their global counterpart. The global output alone is optimized in the Frobenius-attack comparisons. The provided implementation also retains unprojected and partially projected global sketches for the ablation. Tables S1–S5 report the sample-size points, paired comparisons, full-range slopes, projection ablation, and exploratory constant-reference checks, respectively.

Table S1. All five sample-size points used in each clean JPRS slope fit (five seeds per point, $p = 4096$, $s = 128$, $k = 8$). Controlled and high-information sweeps use H1; the theory sweep uses H0. Optional group counts distinguish configuration identity; the global estimator uses all reports.

Sweep	n	Privacy	Groups	Mean loss	95% CI
controlled_n	2000	0.5	7	2.236	[2.038, 2.434]
controlled_n	4000	0.5	7	1.715	[1.561, 1.869]
controlled_n	8000	0.5	7	1.409	[1.22, 1.598]
controlled_n	16000	0.5	7	1.135	[1.069, 1.201]
controlled_n	32000	0.5	7	0.9752	[0.9111, 1.039]
highinfo_n	16000	2	3	0.7033	[0.66, 0.7466]
highinfo_n	32000	2	3	0.4861	[0.4003, 0.5719]
highinfo_n	64000	2	3	0.2362	[0.202, 0.2703]
highinfo_n	128000	2	3	0.07636	[0.06198, 0.09074]
highinfo_n	256000	2	3	0.01498	[0.01079, 0.01916]
theory_n	16000	0.75	7	1.002	[0.9845, 1.02]
theory_n	32000	0.75	7	0.8591	[0.8077, 0.9105]
theory_n	64000	0.75	7	0.6963	[0.6336, 0.7591]
theory_n	128000	0.75	7	0.4635	[0.3902, 0.5367]
theory_n	256000	0.75	7	0.204	[0.1763, 0.2317]

Table S2. Paired JPRS-minus-comparator loss differences (20 seeds), with pointwise 95 percent t intervals. Raw exact two-sided sign-test probabilities and Holm adjustments over all 30 tests are both reported; negative differences favor JPRS.

Scenario	Comparator	Difference	95% CI	Raw p	Holm p
Flat	HR	-0.2613	[-0.297, -0.226]	1.907×10^{-6}	5.722×10^{-5}
Flat	OLH	-0.2431	[-0.272, -0.215]	1.907×10^{-6}	5.722×10^{-5}
Flat	GRR	-6.896	[-7.04, -6.76]	1.907×10^{-6}	5.722×10^{-5}
Flat	Zero	0.5297	[0.519, 0.54]	1.907×10^{-6}	5.722×10^{-5}
Flat	Uniform	0.5453	[0.535, 0.555]	1.907×10^{-6}	5.722×10^{-5}
Zipf	HR	-1.153	[-1.26, -1.05]	1.907×10^{-6}	5.722×10^{-5}
Zipf	OLH	-1.052	[-1.18, -0.922]	1.907×10^{-6}	5.722×10^{-5}
Zipf	GRR	-9.112	[-9.23, -8.99]	1.907×10^{-6}	5.722×10^{-5}
Zipf	Zero	-1.676	[-1.7, -1.65]	1.907×10^{-6}	5.722×10^{-5}
Zipf	Uniform	-1.66	[-1.69, -1.63]	1.907×10^{-6}	5.722×10^{-5}
Community	HR	-0.2703	[-0.293, -0.247]	1.907×10^{-6}	5.722×10^{-5}
Community	OLH	-0.2452	[-0.276, -0.214]	1.907×10^{-6}	5.722×10^{-5}
Community	GRR	-6.908	[-7.09, -6.73]	1.907×10^{-6}	5.722×10^{-5}
Community	Zero	0.5202	[0.511, 0.529]	1.907×10^{-6}	5.722×10^{-5}
Community	Uniform	0.5358	[0.527, 0.545]	1.907×10^{-6}	5.722×10^{-5}
Hub-tail	HR	-0.2999	[-0.346, -0.253]	1.907×10^{-6}	5.722×10^{-5}
Hub-tail	OLH	-0.2731	[-0.299, -0.247]	1.907×10^{-6}	5.722×10^{-5}
Hub-tail	GRR	-6.955	[-7.12, -6.78]	1.907×10^{-6}	5.722×10^{-5}
Hub-tail	Zero	0.4764	[0.453, 0.5]	1.907×10^{-6}	5.722×10^{-5}
Hub-tail	Uniform	0.4921	[0.468, 0.516]	1.907×10^{-6}	5.722×10^{-5}
Mixture	HR	-0.2839	[-0.325, -0.243]	1.907×10^{-6}	5.722×10^{-5}
Mixture	OLH	-0.2428	[-0.268, -0.218]	1.907×10^{-6}	5.722×10^{-5}
Mixture	GRR	-6.902	[-7.04, -6.77]	1.907×10^{-6}	5.722×10^{-5}
Mixture	Zero	0.5016	[0.491, 0.512]	1.907×10^{-6}	5.722×10^{-5}
Mixture	Uniform	0.5172	[0.507, 0.528]	1.907×10^{-6}	5.722×10^{-5}
Variable	HR	-0.4049	[-0.456, -0.354]	1.907×10^{-6}	5.722×10^{-5}
Variable	OLH	-0.3427	[-0.388, -0.297]	1.907×10^{-6}	5.722×10^{-5}
Variable	GRR	-6.965	[-7.08, -6.85]	1.907×10^{-6}	5.722×10^{-5}
Variable	Zero	0.369	[0.341, 0.397]	1.907×10^{-6}	5.722×10^{-5}
Variable	Uniform	0.369	[0.341, 0.397]	1.907×10^{-6}	5.722×10^{-5}

Table S3. Log–log slopes fitted over all five prespecified sample sizes for each seed. Pointwise 95% Student-t intervals summarize five seed-specific slopes; a fitted slope is descriptive, not a proof of a minimax rate.

experiment	method	mean	95% CI
controlled_n	JPRS-Global	-0.2986	[-0.325, -0.272]
controlled_n	P-RS-GRR	0.009023	[-0.0359, 0.054]
controlled_n	P-RS-HR	-0.3324	[-0.366, -0.299]
controlled_n	P-RS-OLH	-0.3469	[-0.412, -0.282]
highinfo_n	JPRS-Global	-1.384	[-1.46, -1.31]
highinfo_n	P-RS-GRR	-0.4478	[-0.527, -0.369]
highinfo_n	P-RS-HR	-0.4441	[-0.457, -0.432]
highinfo_n	P-RS-OLH	-0.737	[-0.775, -0.699]
theory_n	JPRS-Global	-0.5505	[-0.604, -0.497]
theory_n	P-RS-GRR	-0.1729	[-0.2, -0.146]
theory_n	P-RS-HR	-0.2363	[-0.269, -0.204]
theory_n	P-RS-OLH	-0.24	[-0.279, -0.201]

Table S4. Same-global-sketch projection ablation. Entries are mean squared losses over 20 paired seeds per synthetic scenario. Group selection is held out of this comparison.

scenario	Full	Raw	Box only	Top-s + box
flat	1.03	16.45	8.488	3.145
zipf	0.9801	16.6	8.438	2.912
community	1.021	16.27	8.38	3.021
hub_tail	1.151	16.4	8.455	3.187
user_mixture	1.053	16.3	8.404	3.092
variable_cardinality	0.7519	16.33	8.302	2.822

Table S5. Exploratory constant-reference check after inspecting target concentration (512 columns). Each estimator places the fixed constant c in each public other column and zero elsewhere. All five tested constants are shown; none is selected as a tuned method. Entries are deterministic sparse-target losses, outside the 30 primary tests.

dataset	$c = 0$	$c = 0.25$	$c = 0.5$	$c = 0.75$	$c = 1$
Foursquare-NYC-TKY	0.8624	0.9081	1.204	1.749	2.545
Gowalla	3.133	1.245	0.2321	0.09432	0.8316
MovieLens-1M	0.3862	0.4911	1.221	2.576	4.556

S7.2. Attack Search and Decoder Scope

The default contaminated results in the main article use `support_displacement`. Let S_+ be the benchmark target's top-s coordinates and let S_- contain up to s lowest-mass coordinates outside S_+, with randomized ties when choosing S_-. The direction is -1 on S_+, $+1$ on S_-, and zero elsewhere. Each corrupted identity selects a valid report maximizing its linear contribution in this direction to the raw global estimate. For JPRS this is the sign of the inner product of its public pair-sign vector with that direction (random sign on a zero inner product). This targets support ranking; it does not maximize projected squared loss. On real data, "false" denotes coordinates outside the benchmark top-s set and need not mean exactly zero population mass. Target knowledge is available to the evaluation adversary, not to the decoder. The modes `frobenius_sparse` and `frobenius_full` optimize projected squared loss against the sparse and complete targets, respectively. Only exhaustive enumeration establishes a finite-transcript optimum. Bounded coordinate search can stop before one sweep; both its coverage and its budget are recorded. The optimized output is the global feasible decoder. Grouped and ablation branches are evaluated on that same transcript and must not be called their own worst-case attacked outputs. Figure S1 compares objectives and search budgets. No failed scientific result is removed from a completed analysis: execution failures remain in the audit, and the final table includes only a fully rerun schedule.

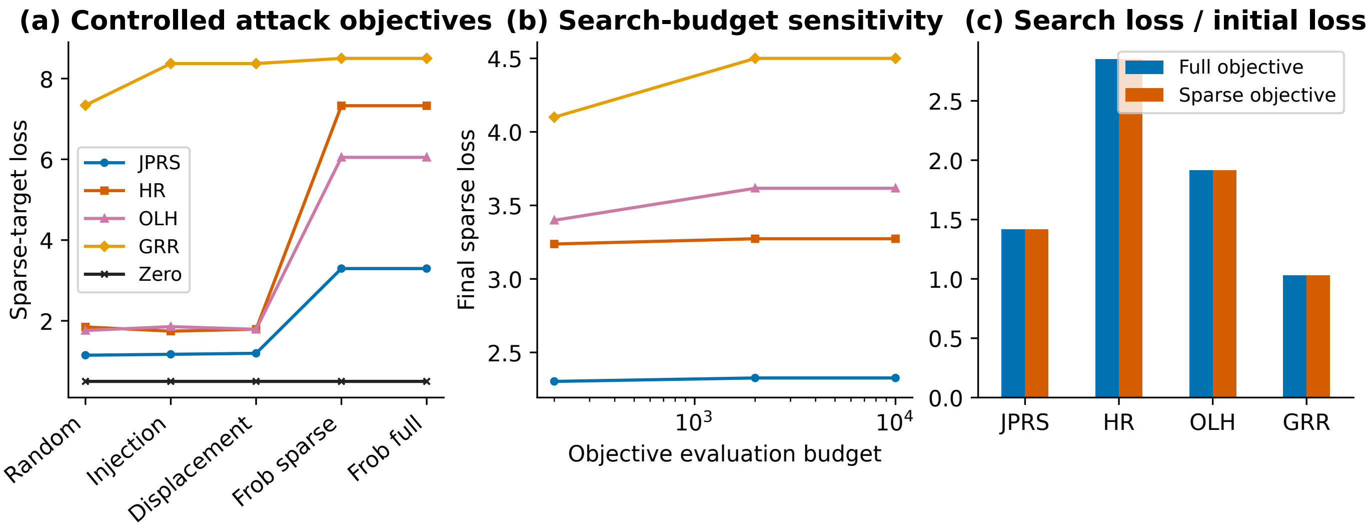

Figure S1. Declared attack objectives and bounded search. Panel (a) uses $n = 16{,}000$, $p = 4096$, $s = 128$, $k = 8$, H1, nominal privacy 0.5, and contamination 0.1. Panel (c) averages the six synthetic families at nominal privacy 1. The budget check in (b) uses $n = 500$, $p = 256$, $s = 32$, $k = 4$, H0, nominal privacy 0.75, and contamination 0.1. All panels show five-seed means without dispersion. Panel (c) compares the final search loss with its displacement initialization; it does not certify global optimality.

Pairwise-uniform hash family.

The OLH hash is $h(j) = b + \sum_{\ell=0}^{L-1} a_\ell \mathrm{bit}_\ell(j) \pmod{g}$, where $L = \lceil \log_2(p+1) \rceil$ and all coefficients are independent uniform elements of $\mathbb{Z}_g$. Distinct labels differ by ± 1 in at least one binary position; that coefficient is invertible modulo every g. Together with the independent intercept this gives pairwise uniform hash outputs and collision probability exactly $1/g$, including composite bucket counts. The experiment simulates these ideal choices using declared pseudorandom streams; it does not prove information-theoretic independence for a deployed finite seed.

S7.3. Sensitivity and Computational Measurements

Figure S2 separates optional group selection, budget heterogeneity, ambient domain size, and dictionary resolution. The complete CSV reports every method and configuration, rather than only favorable endpoints. Parallel scientific-run times are not used as comparative runtime benchmarks. A sequential timing audit was repeated during the consistency review to record its environment contemporaneously: Intel Xeon Platinum 8573C, Linux x86-64 (kernel 6.18.44), eight allocated CPU equivalents, 8 GiB memory, Python 3.12.14, NumPy 2.3.5, pandas 2.2.3, SciPy 1.17.0, and Matplotlib 3.10.8. The runs use one BLAS thread; these timings do not measure deployment latency. A separate sequential audit fixes $n = 16{,}000$, H1, nominal privacy 1, no corruption, seven optional groups, five seeds, one BLAS thread, and $p \in \{1024, 4096, 16384\}$. A family-call time includes shared privatization and all returned grouped outputs; it is not the isolated global decoder's latency. Attack search cost is recorded separately in attacked runs. Private payload excludes authenticated identity and assumed public randomness. Table S6 reports the sequential timing measurements.

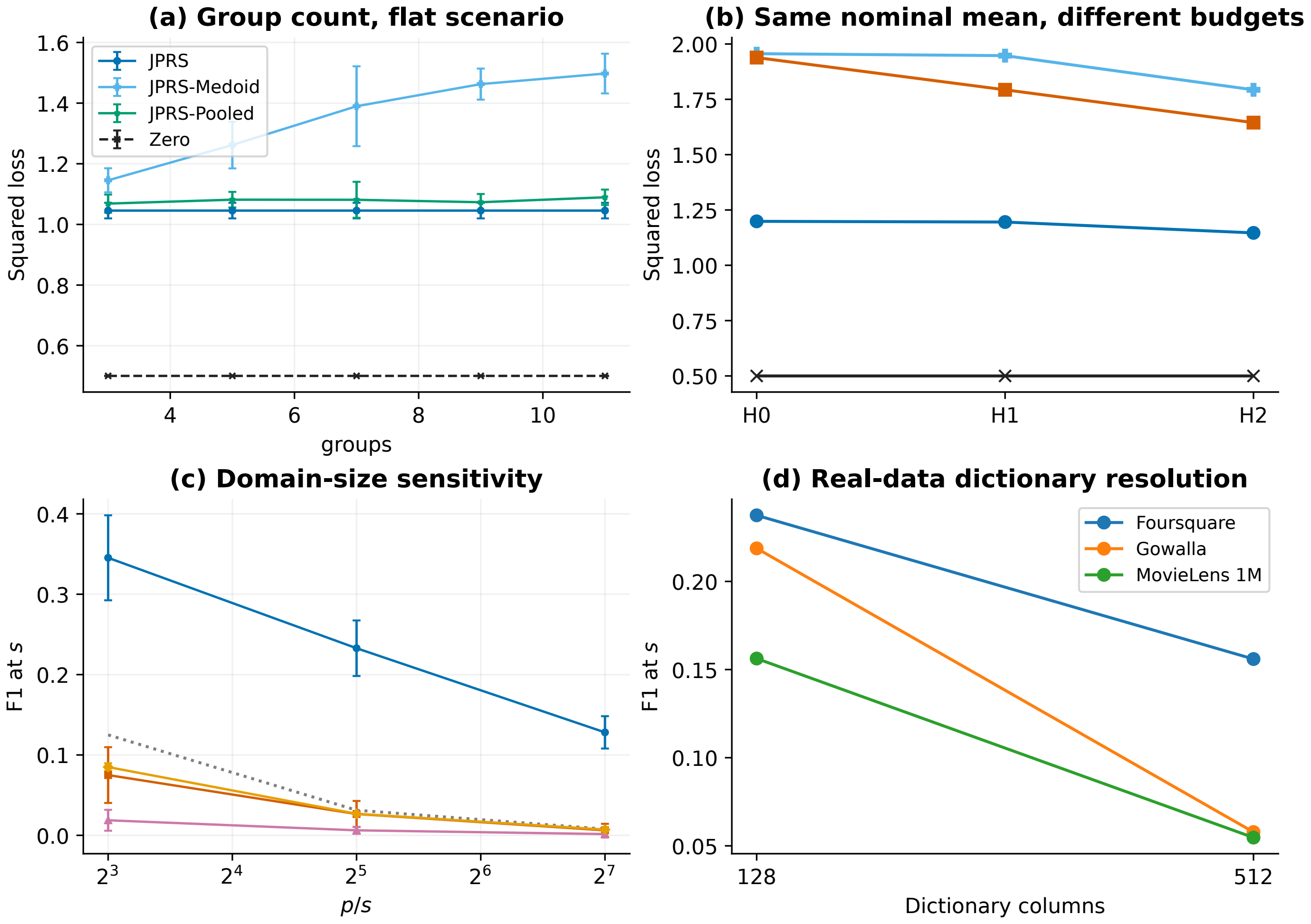

Figure S2. Secondary five-seed diagnostics. The decreasing chance line in (c) prevents direct comparison of unadjusted support scores across domain sizes. Panel (d) changes both dictionary size and the corresponding sparsity budget. Panels (a) and (c) include pointwise 95% Student-*t* CIs for the mean; (b) and (d) show means without dispersion.

S7.4. Primary-Endpoint Regime and Local Slope Diagnostics

The primary endpoint has $n = 16{,}000$, $p = 4096$, $s = 128$, $k = 8$, and $m_\alpha = 1600$. Its balanced H1 profile assigns 8000 identities each to budgets 0.75 and 1.25. Removing 1600

Table S6. Sequential family-call time in seconds (mean and standard deviation over five seeds), repeated with contemporaneous environment recording. One BLAS thread, $n = 16{,}000$, H1, nominal privacy 1, no attack. Payload excludes public randomness and identity metadata.

family	p	seconds	sd	payload
GRR family	1024	0.0374	0.003853	11
GRR family	4096	0.04497	0.01684	13
GRR family	16384	0.06507	0.03028	15
HR family	1024	0.1144	0.005062	1
HR family	4096	0.4644	0.1961	1
HR family	16384	1.794	0.3758	1
JPRS family	1024	0.1629	0.01557	1
JPRS family	4096	0.6052	0.2011	1
JPRS family	16384	2.201	0.3046	1
OLH family	1024	0.3934	0.02552	2
OLH family	4096	1.623	0.09864	2
OLH family	16384	9.363	2.74	2

largest-budget identities leaves 8000 low-budget and 6400 high-budget honest identities. Writing $g_- = \tanh(0.75/2)$ and $g_+ = \tanh(1.25/2)$ gives

$$\mathsf{I}_{\text{eff}}^{(\alpha)} = 8000g_-^2 + 6400g_+^2 = 2995.877653, \qquad \mathsf{L}_{\text{eff}}^{(\alpha)} = \frac{1600g_+}{\mathsf{I}_{\text{eff}}^{(\alpha)}} = 0.296194.$$

The same largest-budget set maximizes leverage: exchanging a smaller selected amplitude for a larger unselected one increases its numerator and decreases its honest-information denominator; adding an identity also increases the ratio. Thus these are the defined worst-set functionals, not mechanism-specific decoder gains or estimates of an average privacy budget. For natural logarithms, the two terms in Corollary S3 before multiplication by its unspecified constant are

$$\frac{ks\log(9epn)}{\mathsf{I}_{\text{eff}}^{(\alpha)}} = 7.244629, \qquad k(\mathsf{L}_{\text{eff}}^{(\alpha)})^2 = 0.701845.$$

Their sum is 7.946474, close to the trivial scale $\bar{k} = 8$, whereas the mean zero-reference losses across the six synthetic families range from 0.382911 to 2.656205. This diagnostic does not certify the regularity condition with an unspecified C_0 or turn the unspecified upper-bound constant into one. It shows why the bound supplies no quantitative guarantee of improvement over the actual zero reference at this endpoint. Failure to improve in five families is consistent with that limitation; it is not a consequence of an impossibility theorem. Although $\mathsf{I}_{\text{eff}}^{(\alpha)} > s^2/k = 2048$, $\max_i \varepsilon_i = 1.25 > 1$, so the strong-privacy matching statement does not apply.

For the clean nominal-privacy-2 H1 sweep, define within each seed r

$$b_{r,j} = \frac{\log L_{s,r}(n_{j+1}) - \log L_{s,r}(n_j)}{\log n_{j+1} - \log n_j}, \qquad n_j \in \{16000, 32000, 64000, 128000, 256000\}.$$

Table S7 reports the five-seed mean of $b_{r,j}$. The progressively steeper slopes document curvature rather than a single power law. The mean full-range least-squares slope remains -1.384; this is a finite-range descriptive summary, not an asymptotic rate. The supplied per-seed values permit both calculations to be reproduced without new runs.

Table S7. Successive clean JPRS log–log slopes in the nominal-privacy-2 H1 sweep. Each entry averages five seed-specific slopes; it is not the slope of the arithmetic mean losses.

Sample-size interval	Mean local slope
16,000–32,000	-0.543
32,000–64,000	-1.038
64,000–128,000	-1.634
128,000–256,000	-2.369

S8. Reproducibility Instructions

The archive contains the immutable full CSV, atomic per-configuration result records, the fixed schedule and seed lists, analysis code, source hashes, confidence intervals, paired tests, attack-search coverage, and build checks. The original 30 synthetic arrays are included; additional endpoint populations are generated by the same released scenario generator at the listed seeds. Raw third-party archives and holder-level derived arrays are not republished. The downloader verifies all three official source hashes before preprocessing; all six processed-file hashes match the supplied original manifest.

Package S1 has two directories. The `evidence/` directory contains frozen results and experiment code; `publication/` contains article sources, figures, and build scripts. From the extracted package root, install the Python dependencies listed in the package README and a LaTeX distribution. The following commands check the archived records, regenerate inspection copies of figures in `publication/rebuilt_assets/`, and compile the manuscripts from the supplied proof sources without rerunning experiments:

```
export PYTHONPATH=evidence/repro
export OPENBLAS_NUM_THREADS=1 OMP_NUM_THREADS=1
python3 publication/check_results.py
python3 publication/replot.py
python3 publication/audit_round2.py --evidence evidence
python3 publication/build.py
python3 publication/check_pdf.py --stage proof
```

To regenerate data, run `python3 -m experiments.restore_real_data` followed by `python3 -m experiments.stage3_run --workers 6` with the same environment. The legacy module name identifies the frozen scientific driver. Existing valid checkpoints are reused; the package README explains full reruns in a separate copy. Repeating the optional sequential timing audit uses `python3 publication/recheck_timing.py`; timings are environment dependent.

The final scientific schedule contains 895 configurations and 12,875 method records with no failures. The initial parallel execution exceeded the available memory and was interrupted. Its failure record is retained. Reducing the computational block from 512 to 64 left the scientific parameters and seed schedule unchanged; the entire schedule was rerun. The implementation/configuration hash prevents mixing those attempts. Separately, 30 planned real-attack jobs inherited an unintended 64-column dictionary default. Eighteen have recorded file-loading failures, before any estimates were produced. The other 12 (seeds 4409 and 5519, each with three datasets and two attack objectives) have no retained completion record under that configuration. The retained records contain no successful numerical results for any of these 30 old configurations; in particular, they do not document 12 successful 64-column runs that were discarded. The records do not distinguish whether these 12 jobs had not started or were interrupted before completion. All 30 planned configurations were corrected to 512 columns and completed successfully. This dictionary correction is distinct from the earlier computational-block reduction. After an execution-

session interruption, 483 successful configurations were retained with matching hashes and the remaining 412 were completed. No failed job supplies a numerical loss. The supplementary audit script writes a 30-row job ledger with old and corrected job identifiers, recorded outcomes, and final completion status, along with the endpoint calculation and per-seed local slopes in `publication/analysis/`. The final audit distinguishes completed scientific configurations, rerun failures, and sequential timing measurements. Tests cover projection optimality, protocol boundaries, legal attacks, tie-averaged metrics, paired tests, and multiplicity correction. Five-seed diagnostic intervals remain approximate.

References

1. Bassily, R.; Smith, A. Local, Private, Efficient Protocols for Succinct Histograms. In Proceedings of the 47th Annual ACM Symposium on Theory of Computing. Association for Computing Machinery, 2015, pp. 127–135. https://doi.org/10.1145/2746539.2746632.
2. Shah, A.; Chen, W.N.; Ballé, J.; Kairouz, P.; Theis, L. Optimal Compression of Locally Differentially Private Mechanisms. In Proceedings of the 25th International Conference on Artificial Intelligence and Statistics. PMLR, 2022, Vol. 151, *Proceedings of Machine Learning Research*, pp. 7680–7723. https://proceedings.mlr.press/v151/shah22b.html.
3. Cao, X.; Jia, J.; Gong, N.Z. Data Poisoning Attacks to Local Differential Privacy Protocols. In Proceedings of the 30th USENIX Security Symposium. USENIX Association, 2021, pp. 947–964. https://www.usenix.org/conference/usenixsecurity21/presentation/cao-xiaoyu.
4. Wu, Y.; Cao, X.; Jia, J.; Gong, N.Z. Poisoning Attacks to Local Differential Privacy Protocols for Key-Value Data. In Proceedings of the 31st USENIX Security Symposium. USENIX Association, 2022, pp. 519–536. https://www.usenix.org/conference/usenixsecurity22/presentation/wu-yongji.
5. Li, X.; Li, N.; Sun, W.; Gong, N.Z.; Li, H. Fine-Grained Poisoning Attack to Local Differential Privacy Protocols for Mean and Variance Estimation. In Proceedings of the 32nd USENIX Security Symposium. USENIX Association, 2023, pp. 1739–1756. https://www.usenix.org/conference/usenixsecurity23/presentation/li-xiaoguang.
6. Duchi, J.C.; Jordan, M.I.; Wainwright, M.J. Minimax Optimal Procedures for Locally Private Estimation. *Journal of the American Statistical Association* **2018**, *113*, 182–201. https://doi.org/10.1080/01621459.2017.1389735.
7. Kairouz, P.; Oh, S.; Viswanath, P. Extremal Mechanisms for Local Differential Privacy. *Journal of Machine Learning Research* **2016**, *17*, 1–51. https://jmlr.org/papers/v17/15-135.html.
8. Huber, P.J. Robust Estimation of a Location Parameter. *The Annals of Mathematical Statistics* **1964**, *35*, 73–101. https://doi.org/10.1214/aoms/1177703732.
9. Chhor, J.; Sentenac, F. Robust Estimation of Discrete Distributions under Local Differential Privacy. In Proceedings of the 34th International Conference on Algorithmic Learning Theory. PMLR, 2023, Vol. 201, *Proceedings of Machine Learning Research*, pp. 411–446. https://proceedings.mlr.press/v201/chhor23a.html.
10. Wang, S.; Li, J.; Qian, Y.; Du, J.; Lin, W.; Yang, W. Hiding Numerical Vectors in Local Private and Shuffled Messages. In Proceedings of the Thirtieth International Joint Conference on Artificial Intelligence, 2021, pp. 3706–3712. https://doi.org/10.24963/ijcai.2021/510.
11. Zhou, M.; Wang, T.; Chan, T.H.H.; Fanti, G.; Shi, E. Locally Differentially Private Sparse Vector Aggregation. In Proceedings of the 2022 IEEE Symposium on Security and Privacy. IEEE, 2022, pp. 422–439. https://doi.org/10.1109/SP46214.2022.9833635.
12. Acharya, J.; Sun, Z.; Zhang, H. Hadamard Response: Estimating Distributions Privately, Efficiently, and with Little Communication. In Proceedings of the 22nd International Conference on Artificial Intelligence and Statistics. PMLR, 2019, Vol. 89, *Proceedings of Machine Learning Research*, pp. 1120–1129. https://proceedings.mlr.press/v89/acharya19a.html.
13. Wang, T.; Blocki, J.; Li, N.; Jha, S. Locally Differentially Private Protocols for Frequency Estimation. In Proceedings of the 26th USENIX Security Symposium. USENIX Association, 2017, pp. 729–745. https://www.usenix.org/conference/usenixsecurity17/technical-sessions/presentation/wang-tianhao.

Disclaimer/Publisher's Note: The statements, opinions and data contained in all publications are solely those of the individual author(s) and contributor(s) and not of MDPI and/or the editor(s). MDPI and/or the editor(s) disclaim responsibility for any injury to people or property resulting from any ideas, methods, instructions or products referred to in the content.
