# Supplementary material for: Information-Theoretic Limits and One-Bit Estimation of Sparse Relation Matrices Under Personalized Local Differential Privacy and Byzantine Contamination
Source: Entropy (Basel). 2026 Oct 5;28(10):1088. doi: 10.3390/e28101088 (PMC13703432; doi:10.3390/e28101088)
Supplement: Supplementary file 1 [file entropy-28-01088-s001.zip › entropy-4532751-supplementary/Package_S1.zip!/Package_S1/publication/rebuilt_assets/figures/fig_controlled.pdf]

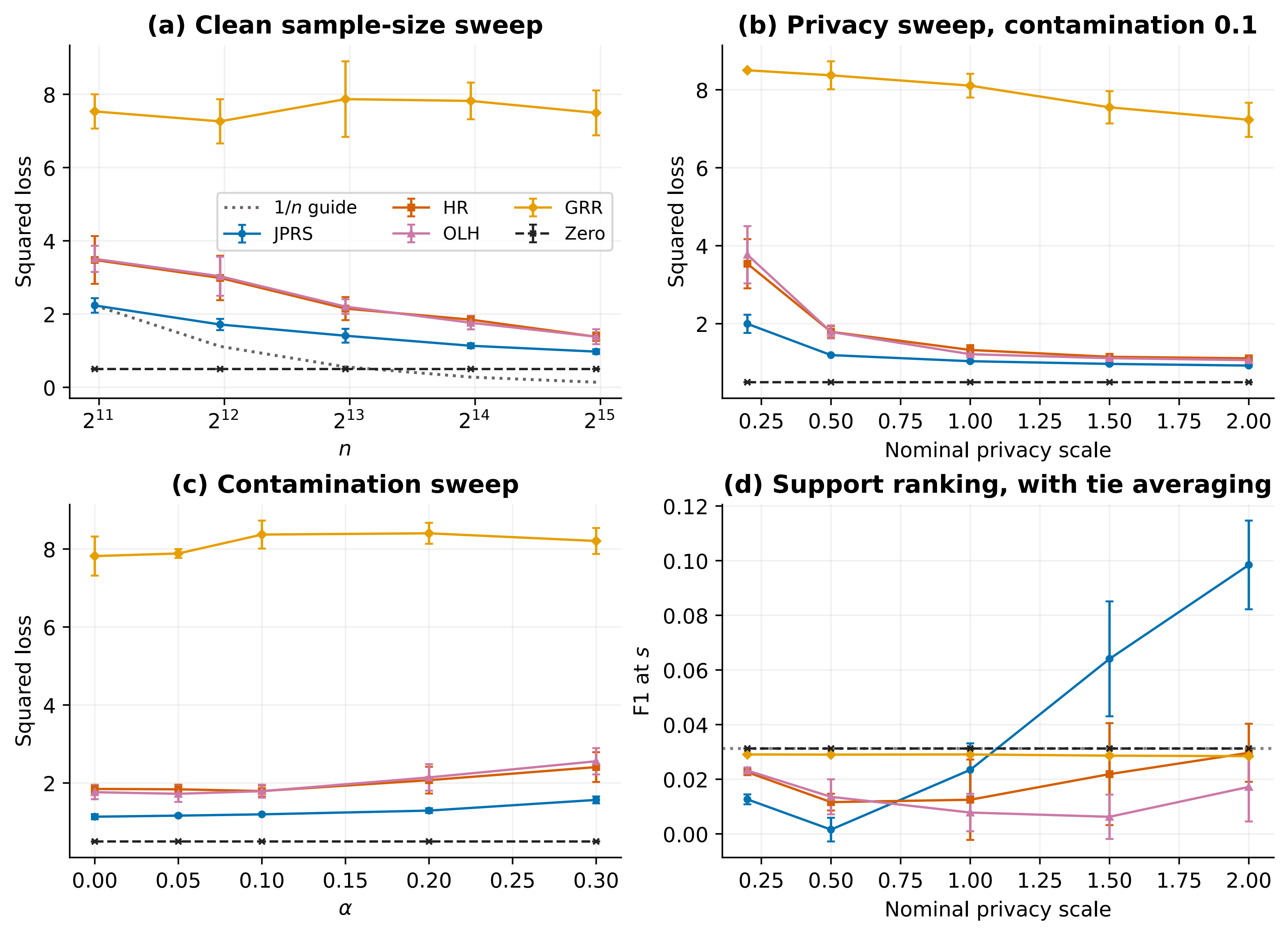

(a) Clean sample-size sweep
Squared loss
n
1/n guide
JPRS
HR
OLH
GRR
Zero
(b) Privacy sweep, contamination 0.1
Squared loss
Nominal privacy scale
(c) Contamination sweep
Squared loss
α
(d) Support ranking, with tie averaging
F1 at s
Nominal privacy scale
