# Supplementary material for: Information-Theoretic Limits and One-Bit Estimation of Sparse Relation Matrices Under Personalized Local Differential Privacy and Byzantine Contamination
Source: Entropy (Basel). 2026 Oct 5;28(10):1088. doi: 10.3390/e28101088 (PMC13703432; doi:10.3390/e28101088)
Supplement: Supplementary file 1 [file entropy-28-01088-s001.zip › entropy-4532751-supplementary/Package_S1.zip!/Package_S1/publication/rebuilt_assets/figures/fig_synthetic.pdf]

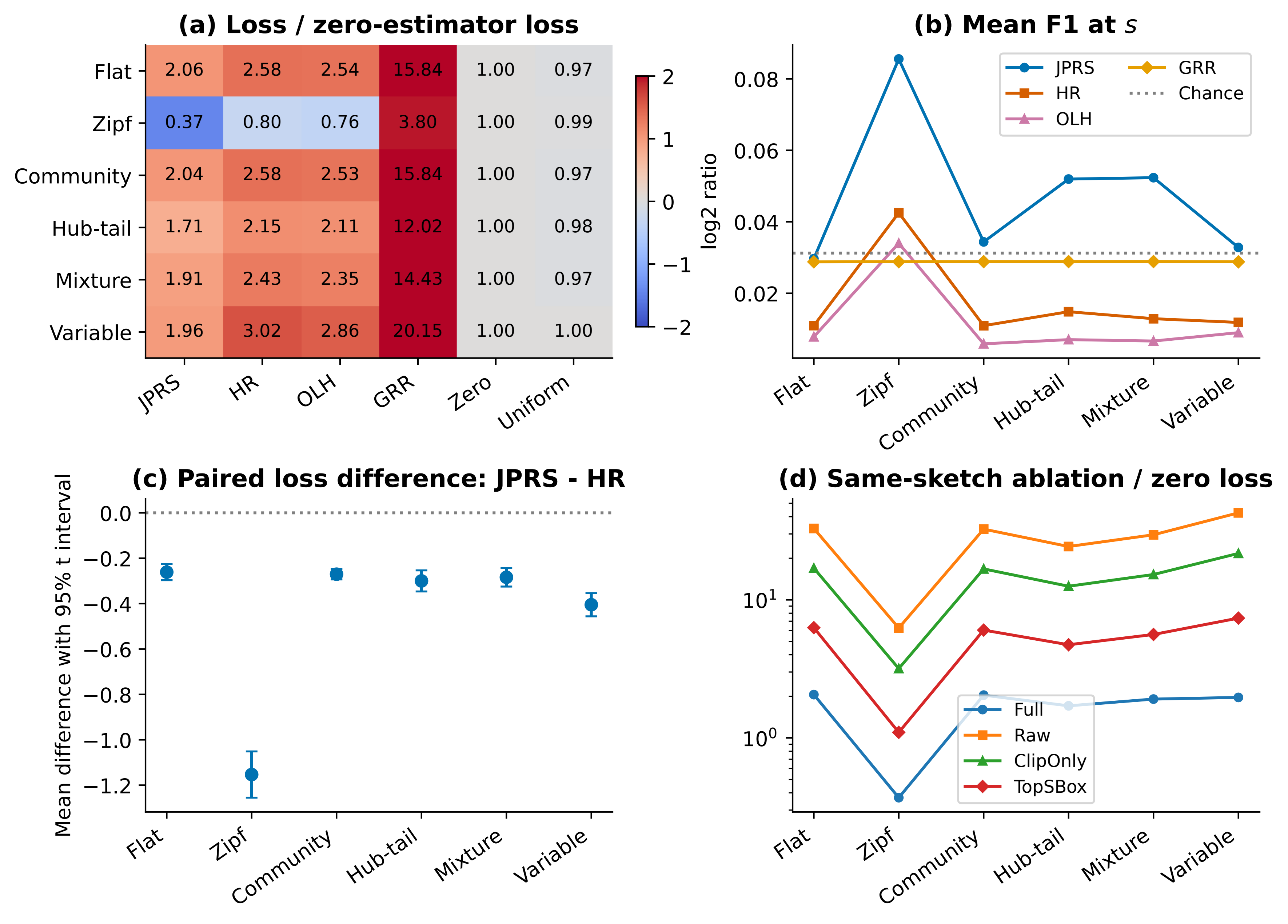

(a) Loss / zero-estimator loss
Flat
Zipf
Community
Hub-tail
Mixture
Variable
2.06 2.58 2.54 15.84 1.00 0.97
0.37 0.80 0.76 3.80 1.00 0.99
2.04 2.58 2.53 15.84 1.00 0.97
1.71 2.15 2.11 12.02 1.00 0.98
1.91 2.43 2.35 14.43 1.00 0.97
1.96 3.02 2.86 20.15 1.00 1.00
JPRS
HR
OLH
GRR
Zero
Uniform
log2 ratio
2
1
0
−1
−2
(b) Mean F1 at s
JPRS
HR
OLH
GRR
Chance
0.08
0.06
0.04
0.02
Flat
Zipf
Community
Hub-tail
Mixture
Variable
(c) Paired loss difference: JPRS - HR
Mean difference with 95% t interval
0.0
−0.2
−0.4
−0.6
−0.8
−1.0
−1.2
Flat
Zipf
Community
Hub-tail
Mixture
Variable
(d) Same-sketch ablation / zero loss
Full
Raw
ClipOnly
TopSBox
10^1
10^0
Flat
Zipf
Community
Hub-tail
Mixture
Variable
